# Cyclin B1 stability is increased by interaction with BRCA1, and its overexpression suppresses the progression of BRCA1-associated mammary tumors

**DOI:** 10.1038/s12276-018-0169-z

**Published:** 2018-10-16

**Authors:** Eun Kyung Choi, Jeong-A Lim, Jong Kwang Kim, Moon Sun Jang, Sun Eui Kim, Hye Jung Baek, Eun Jung Park, Tae Hyun Kim, Chu-Xia Deng, Rui-Hong Wang, Sang Soo Kim

**Affiliations:** 1 Research Institute, National Cancer Center, Goyang, 10408 Korea; 2 Cancer Centre, Faculty of Health Sciences, University of Macau, Macau, 999078 China

## Abstract

Germline *BRCA1* mutations predispose women to breast and ovarian cancer. BRCA1, a large protein with multiple functional domains, interacts with numerous proteins involved in many important biological processes and pathways. However, to date, the role of BRCA1 interactions at specific stages in the progression of mammary tumors, particularly in relation to cell cycle regulation, remains elusive. Here, we demonstrate that BRCA1 interacts with cyclin B1, a crucial cell cycle regulator, and that their interaction is modulated by DNA damage and cell cycle phase. In DNA-damaged mitotic cells, BRCA1 inhibits cytoplasmic transportation of cyclin B1, which prevents cyclin B1 degradation. Moreover, restoration of cyclin B1 in BRCA1-deficient cells reduced cell survival in association with induction of apoptosis. We further demonstrate that treatment of *Brca1*-mutant mammary tumors with vinblastine, which induces cyclin B1, significantly reduced tumor progression. In addition, a correlation analysis of vinblastine responses and gene expression profiles in tumors at baseline revealed 113 genes that were differentially expressed between tumors that did and did not respond to vinblastine treatment. Further analyses of protein–protein interaction networks revealed gene clusters related to vinblastine resistance, including nucleotide excision repair, epigenetic regulation, and the messenger RNA surveillance pathway. These findings enhance our understanding of how loss of BRCA1 disrupts mitosis regulation through dysregulation of cyclin B1 and provide evidence suggesting that targeting cyclin B1 may be useful in BRCA1-associated breast cancer therapy.

## Breast cancer: A protein link for treatment?

The role of disrupted activity of the protein BRCA1 in the progression of breast cancer has been clarified, suggesting that targeting another protein with which it interacts could offer a new route to treatment. Mutations of *BRCA1* are known to predispose women to both breast and ovarian cancers. Researchers led by Sang Soo Kim (National Cancer Center, South Korea) and Rui-Hong Wang (University of Macau, China) studied the interaction with a protein called cyclin B1 that controls cell growth and division. They found that, in mitosis, BRCA1 interacts with and stabilizes cyclin B1, explaining why the loss of BRCA1 can disrupt the G2/M cell cycle control and accumulate the genetic instability. Treatment of *Brca1*-mutant mammary tumors with vinblastine, which alters cyclin B1 level, significantly reduced tumor progression with reduction of survival and induction of apoptosis.

## Introduction

The BRCA1 protein is a tumor suppressor that has a crucial role in maintenance of genomic integrity. Inherited mutations in the *BRCA1* gene predispose women to early-onset breast and ovarian cancers^1,2^. In *BRCA1* carrier women, *BRCA1* mutations result in a 57–65% risk of developing breast cancer and a 39–44% risk of developing ovarian cancer by age 70 years^3^. Previous investigations of BRCA1 have suggested that the multifunctional role of BRCA1 is attributable to interactions in various cellular compartments with different protein partners that play essential roles in diverse cellular pathways, including DNA damage repair, cell cycle checkpoint regulation, centrosome duplication, and apoptosis^4,5^.

BRCA1 has been consistently linked to control of cell cycle and has been shown to induce arrest at several cell cycle phases, a function that would appear to complement its role in DNA damage repair processes by allowing adequate time for DNA repair to occur. Deregulation of cell cycle control, which enables cells with acquired genomic alterations to proliferate, is frequently identified in BRCA1-associated breast cancer^6^. During cell cycle progression, BRCA1 protein undergoes hyperphosphorylation in late G_1_ and S phase and is transiently dephosphorylated early after M phase^7^. Notably, BRCA1 is phosphorylated by the serine/threonine kinase ATM (ataxia telangiectasia mutated) in the context of DNA damage, and its phosphorylation at Ser1387 and Ser1423 is required for S-phase and G2/M-phase checkpoints, respectively^8,9^. In addition, Aurora-A kinase physically binds and phosphorylates BRCA1 at Ser308, a phosphorylation that is correlated with impaired BRCA1-mediated regulation of G_2_/M transition^10^. Chk2, a substrate of ATM, phosphorylates Ser988 of BRCA1 and induces the release of BRCA1 from Chk2, thereby allowing survival after recovery from DNA damage^11^. Mouse embryo fibroblasts (MEFs) generated from *Brca1*^*S971A/S971A*^ embryos containing the equivalent mouse *Brca1* mutation (Ser971) exhibit a partial loss of the G_2_/M cell cycle checkpoint upon irradiation, suggesting that BRCA1 regulation of the G_2_/M checkpoint is partially modulated by Chk2 phosphorylation^12^.

BRCA1 is also associated with numerous proteins that have been implicated in important functions in all cell cycle phases, and its deficiency consequently causes abnormalities in checkpoint control. Aprelikova et al.^13^ reported that BRCA1 induces G_1_ arrest in the presence of RB (retinoblastoma protein) and further showed that BRCA1 interacts with hypophosphorylated RB. Since hypophosphorylated RB interacts with the transcription factor E2F to prevent transcription of downstream genes, thereby inhibiting cell proliferation, it is conceivable that binding to BRCA1 maintains RB in the hypophosphorylated state necessary to achieve growth arrest. BRCA1 also interacts with several proteins that play essential roles in the S-phase checkpoint, including MDC1 (mediator of DNA damage checkpoint protein 1), H2AFX (H2A histone family member X), 53BP1 (p53 binding protein 1), and MRN (MRE11/RAD50/NBS1), which form nuclear foci in response to ionizing radiation and cause cell cycle arrest in the S phase^14^. In addition, it has been shown that BRCA1 associates with Cdk1 (cyclin-dependent kinase-1), Cdk2 and Cdk4, cyclin B, cyclin D, cyclin A, and the transcription factor E2F4 but not with Cdk3, Cdk5, Cdk6, E2F1, E2F2, E2F3, E2F5, or cyclin E. These observations suggest that BRCA1 could be an important negative regulator of cell cycle^15^.

Among BRCA1-interacting proteins, cyclin B1 has been reported to exhibit inconsistencies in terms of its crosstalk with BRCA1. In BRCA1-deficient tumor cells, cyclin D1 is stabilized, and other cyclins, including cyclin A, cyclin B1, and cyclin E, are undetectable^16^. In addition, *Brca1*^*Δ11/Δ11*^-mutant cells and cells with a 4-hydroxytamoxifen (4-HT)-induced deletion of BRCA1 showed a decrease in cyclin B1 and histone H1 kinase levels^17^. However, overexpression of BRCA1 has also been reported to reduce the level of cyclin B1 and decrease the activity of histone H1 kinase^18^. A more recent study showed that BRCA1 ubiquitinates the G2/M cell cycle proteins cyclin B and Cdc25C, leading to their accelerated degradation via a mechanism that is independent of anaphase-promoting complex/cyclosome (APC/C)^19^.

Here, to identify the role of BRCA1 in DNA damage, we analyzed BRCA1-interacting proteins using the BRCA1-Δ11 isoform, which does not exhibit cytotoxic effects upon overexpression. Our biochemical analyses identified cyclin B1 as the protein that showed the most prominent increase in its interaction with BRCA1-Δ11 upon irradiation. We further characterized the interaction of BRCA1 and cyclin B1 and performed preclinical tests of vinblastine, a cyclin B1-inducing agent, in the treatment of BRCA1-associated mammary tumors using a *Brca1*-mutant allograft model.

## Materials and methods

### Mice and tumor allografts

*Brca1* conditional-knockout mice and *MMTV-Cre* transgenic mice were provided by the National Cancer Institute Mouse Repository (Frederick, MD, USA). Female *Brca1*-mutant mice were generated by crossing *Brca1* conditional-knockout mice with *MMTV-Cre* mice, which were originally generated by Drs. Deng and Hennighausen, respectively^20,21^. For tumor allografts, spontaneously developed primary tumors obtained from eight tumor-bearing *Brca1*^*co/co*^*MMTV-Cre* mice were orthotopically implanted into 4-week-old female HsdCpb:NMRI-*Foxn1*^*nu*^ mice (Orient-Harlan Laboratories, Seongnam, Korea). After each grafted tumor reached ~1000 mm^3^, the tumor tissue was excised, trimmed with a tissue slicer, and reimplanted into recipient mice. Beginning 1 week after implantation, recipient mice were treated with vehicle or vinblastine (0.5 mg/kg, 5 times per week, injected intraperitoneally). Tumor size (length and width, in mm) was measured at least twice a week from the initial treatment using calipers, and tumor volume (in mm^3^) was calculated according to the following formula: *V* = 0.5 × *d*^2^ × *D*, where *d* is the shorter diameter and *D* is the longer diameter. Tumor growth was assessed as the ratio of the tumor volume (RTV) at a given time to that recorded at the initiation of treatment (baseline tumor); assessments were made until the tumor volume reached ~3000 mm^3^. Vinblastine sulfate (Abmole Bioscience, Houston, TX, USA) was dissolved at a concentration of 50 mg/mL in phosphate-buffered saline. All procedures involving animals and their care were approved by the Institutional Animal Care and Use Committee of the National Cancer Center of Korea.

### Cell culture and analysis

MEFs were derived from embryonic day 14.5 embryos generated from intercrosses of *Brca1*^*+/Δ11*^ animals. All comparisons were made between *Brca1*^*Δ11/Δ11*^-mutant mice and their wild-type (WT) *Brca1*^*+/+*^ littermates. For acute deletion of *Brca1*, MEFs from *Brca1* conditional-mutant (*Brca1*^*co/co*^) embryos were infected with an adenovirus-expressing Cre-recombinase (Ad-Cre) together with internal ribosome entry site-green fluorescent protein (IRES-GFP) (Ad-Cre; Vector Biolabs, Malvern, PA, USA). For comparison, MEFs expressing WT *Brca1* were infected with an adenovirus-expressing IRES-GFP only (Ad-GFP; Vector Biolabs). Deletion of *Brca1* was confirmed by monitoring GFP expression under a fluorescence microscope and by Western blotting. MCF7 and 293T cell lines were purchased from American Type Culture Collection (Manassas, VA, USA) and the Korean Cell Line Bank (Seoul, Korea), respectively. The authenticity of human cell lines was confirmed by short tandem repeat analysis performed by the Omics Core of the National Cancer Center. The *Brca1*-mutant mouse cell lines were as described previously^22^.

Several variants of *CCNB1* and *BRCA1* were cloned into *pCAGG-FLAG or pcDNA3.1/V5-His* plasmids (ThermoFisher, Waltham, MA, USA) using polymerase chain reaction (PCR). *BRCA1* expression was knocked down by transfecting cells with *BRCA1*-targeting small interfering RNAs (siRNAs) (Santa Cruz, Dallas, TX, USA) using Lipofectamine 2000 (ThermoFisher); scrambled small interfering RNA (siRNA) (Dharmacon, Lafayette, CO, USA) was used as a control. For overexpression of cyclin B1, cells were infected with an adenovirus-expressing cyclin B1 (Ad-cyclin B1; Vector Biolabs); Ad-GFP was used as a control.

For cell growth monitoring, cells were plated (in quadruplicate) at 2 × 10^4^ cells per well in 4-well plates and treated as indicated, after which the cell viability was determined using an MTT (3-(4,5-dimethylthiazol-2-yl)-2,5-diphenyltetrazolium bromide)-based In Vitro Toxicology Assay Kit (Sigma, St. Louis, MO, USA) according to the manufacturer’s instructions. A flow cytometry analysis of mitotic index and DNA content was performed as described previously^12^ using a FACSCalibur flow cytometer and CellQuest (BD Biosciences, San Jose, CA, USA) analysis software.

### Western blotting, immunoprecipitation, and confocal microscopy

Western blot analysis was carried out according to standard procedures using enhanced-chemiluminescence detection (GE Healthcare, Chicago, IL, USA). Tumor tissue lysates were prepared using an electric homogenizer for 30 s after the addition of lysis buffer. The following antibodies were used: anti-β-actin, anti-caspase-3, anti-caspase-7, anti-phospho-CDC20, anti-cyclin D1, anti-histone H3, anti-PARP, and anti-phospho-Rb (Cell Signaling Technology, Danvers, MA, USA); anti-β-actin, anti-BRCA1, anti-CDC20, anti-cyclin B1, anti-cyclin D1, anti-Myc, anti-p53, and anti-α-tubulin (Santa Cruz); anti-FLAG (Sigma); anti-p21 (BD Pharmingen, San Jose, CA, NJ, USA); anti-phospho-histone H3 (Merck Millipore, Darmstadt, Germany); and anti-proliferating cell nuclear antigen (PCNA; Atlas Antibodies, Bromma, Sweden). Horseradish peroxidase-conjugated goat anti-rabbit or anti-mouse antibodies (Jackson Immuno Research, West Grove, PA, USA) were used as secondary antibodies, as appropriate.

For immunoprecipitation, lysates (1 mg protein) of cells transfected with different variants of BRCA1 and/or cyclin B1 were incubated with 1 μg of primary antibody and then further incubated overnight at 4 °C with 20 μL of protein A/G Sepharose beads (GE Healthcare). Immunoprecipitates were then eluted with the sample buffer (50 mmol/L Tris (pH 7.5), 1% sodium dodecyl sulfate, 100 mmol/L dithiothreitol). For immunoprecipitation of FLAG-tagged proteins, cell lysates were incubated with an anti-FLAG M2 affinity gel (Sigma) and eluted with 4× FLAG peptides (Sigma).

For cell staining, MCF7 cells were stained with anti-cyclin B1 immunoglobulin G (IgG) (Cell Signaling Technology) and anti-BRCA1 IgG (Santa Cruz) and observed under a Zeiss Axiovert LSM780 microscope (Carl Zeiss, Oberkochen, Germany).

### Histology and immunohistochemistry

For histology, tissues were fixed in 10% (v/v) formalin, embedded in paraffin, sectioned, stained with hematoxylin and eosin, and examined by light microscopy. Antigenic proteins were detected using a Zymed HistoMouse SP Kit (ThermoFisher) according to the manufacturer’s instructions. The primary antibodies used were anti-phospho-histone H3 (Merck Millipore) and anti-PCNA (Atlas Antibodies). Apoptotic cells were assessed using TUNEL (terminal deoxynucleotidyl transferase dUTP nick-end labeling) assays (Merck Millipore), and the accumulation of collagen in tissues was monitored using Masson’s trichrome staining (Sigma). All the histochemical analysis comparisons were performed in a manual tissue microarray (Unitma, Seongnam, Korea) containing control and treated samples within the same slide.

### Expression analysis

Tumor tissues were dissected free of their surrounding normal tissues and immediately frozen in a pre-chilled aluminum block. Total RNA was purified from tumor tissues using an RNeasy Mini Kit (Qiagen, Hilden, Germany) and subjected to genome-wide RNAseq analyses, performed by eBiogene (Seoul, Korea). Raw data obtained from duplicate analyses of eight samples were normalized using Cufflinks RNAseq workflow^23^. Spearman’s rank correlation (*⍴* > 0.7, *P* *<* 0.01) was performed to select genes that were correlated with the RTV. Highly correlated genes (HCGs) were selected as markers, and a heat map was generated using the *z*-scores of their normalized expression in fragments per kilobase per million mapped fragments (FPKMs). Samples were sorted according to the correlation of their RTV with their gene expression pattern. Heat maps were generated using normalized FPKMs of HCGs identified in each adjuvant-based experiment as input to the heat map function of the Superheat R open source package. Spearman’s rank correlation (http://sites.utexas.edu/sos/guided/inferential/numeric/bivariate/rankcor/) was used to correlate gene expression with tumor volume, as specified in the text. A *P* value <0.01 was considered statistically significant.

### Functional network analysis

All known interactions between molecules that interacted with our markers were analyzed using Search Tool for the Retrieval of Interacting Genes/proteins (STRING), a database of known and predicted protein–protein interactions. Direct and indirect associations yielded by computational predictions and interactions obtained from other interaction analysis databases (https://www.string-db.org) were included^24,25^. The STRING score is calculated from the combination of all predictions (range, 0–1) and classified into four categories: highest confidence (0.900), high confidence (0.700), medium confidence (0.400), and low confidence (0.150). For network analysis, confidence was set at high (STRING score = 0.7). Only experimentally determined interactions and known interactions from curated databases were included in our analysis. Nodes in the network were grouped according to enriched Kyoto Encyclopedia of Genes and Genomes (KEGG) pathways (false discovery rate <0.05).

### Statistical analysis

Student’s *t* test (http://www.physics.csbsju.edu/stats/t-test.html) was used to compare differences in means between two groups as specified in the text. A *P* value <0.05 was considered statistically significant.

## Results

### BRCA1 interacts with cyclin B1

**Fig. 1:**
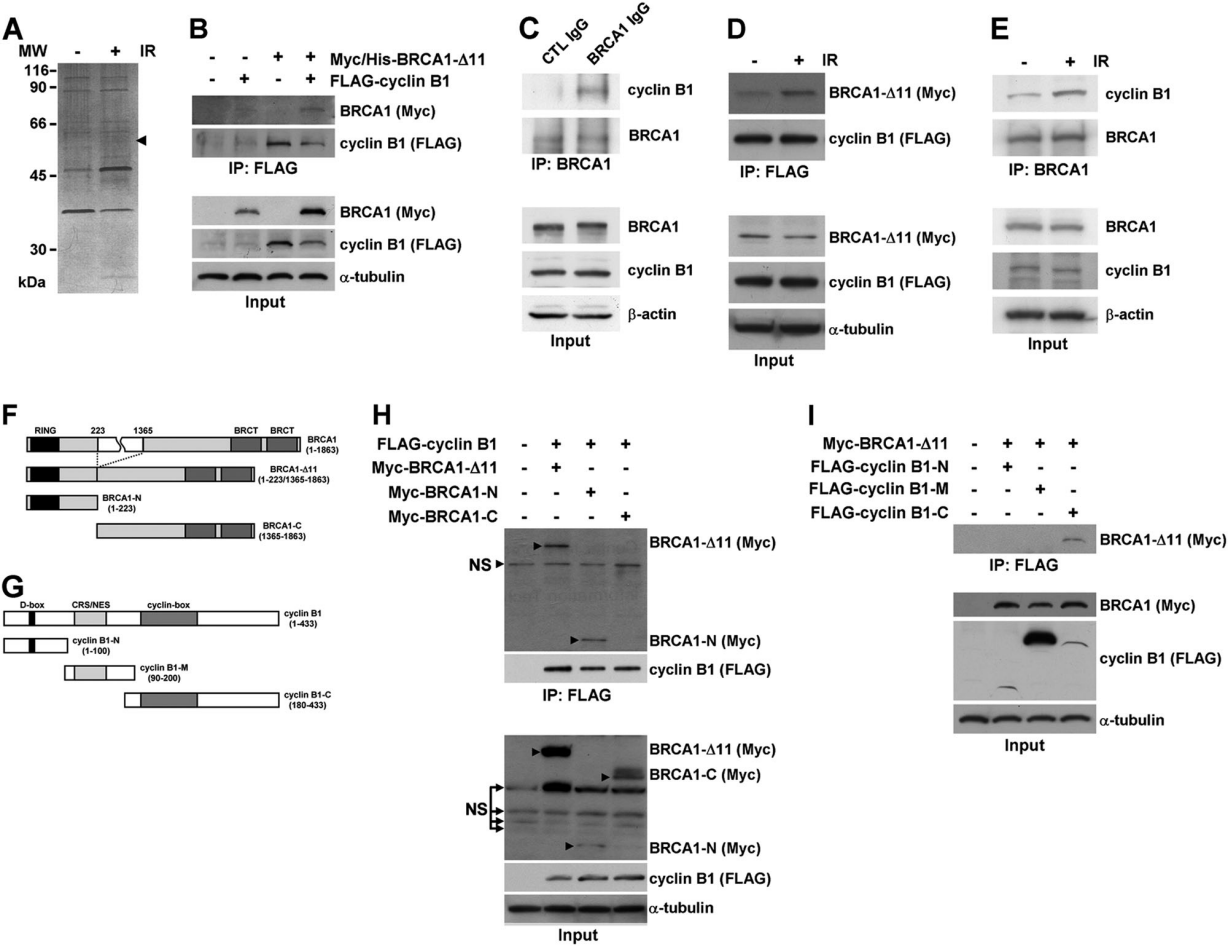
BRCA1 interacts with cyclin B1. **a** MCF7 cells were transfected with a construct encoding His-tagged BRCA-Δ11 and irradiated to induce DNA damage. Extracts of transfected MCF7 cells were prepared and subjected to Ni-NTA affinity chromatography, and the resulting eluates were analyzed by sodium dodecyl sulfate (SDS)-polyacrylamide gel electrophoresis and silver staining. The arrow indicates cyclin B1 further determined by matrix -assisted laser desorption ionization-time of flight (MALDI-TOF) mass spectrometry. **b** Lysates of 293T cells transfected with Myc-BRCA-Δ11 and/or FLAG-cyclin B1 were immunoprecipitated with anti-FLAG antibody-conjugated agarose. The resulting precipitates as well as the original lysates (Input) were analyzed by immunoblotting with the indicated antibodies. **c** Lysates of irradiated MCF7 cells were immunoprecipitated with control or anti-BRCA1 IgG, followed by Western blot analyses using the indicated antibodies. **d** The 293T cells transfected with Myc-BRCA1-Δ11 and FLAG-cyclin B1 were irradiated (10 Gy) and immunoprecipitated with anti-FLAG antibody-conjugated agarose, after which interactions were assessed by Western blot analysis of immunoprecipitates using the indicated antibodies. **e** Lysates of MCF7 cells in the absence or presence of irradiation were immunoprecipitated with an antibody against BRCA1, followed by Western blot analyses using the indicated antibodies. **f** Myc fusion constructs of BRCA1-Δ11 and deletion mutants. RING Really Interesting New Gene finger domain, BRCT BRCA1 C-terminal domain. **g** FLAG fusion constructs of cyclin B1 and deletion mutants. D-box destruction box domain CRS/NES, cytoplasmic retention signal/nuclear export signal domain. **h** Lysates of 293T cells transfected with FLAG-cyclin B1, Myc-BRCA1-Δ11, Myc-BRCA1 N-terminal fragment, and/or Myc-BRCA1 C-terminal fragment constructs were immunoprecipitated with anti-FLAG antibody-conjugated agarose and analyzed by immunoblot analysis with the indicated antibodies. **i** Lysates of 293T cells transfected with Myc-BRCA1-Δ11 or several forms of FLAG-cyclin B1 constructs were immunoprecipitated with anti-FLAG antibody-conjugated agarose, and immunoprecipitates were analyzed by immunoblotting using the indicated antibodies. Input represents 5% of the protein extract prior to immunoprecipitation

Numerous BRCA1-interacting proteins have been identified using antibody-based assays and yeast two-hybrid screens. However, it is unclear how many of these proteins form complexes with BRCA1 in diverse cellular events. To approach this question in the context of DNA damage, we investigated BRCA1-interacting proteins using the BRCA1-Δ11 isoform, which, unlike full-length BRCA1 isoforms, does not exhibit cytotoxicity upon overexpression, enabling expression of the protein at high levels. MCF7 cells were transfected with a construct encoding His-tagged BRCA-Δ11 and irradiated to induce DNA damage. Proteins that interacted with BRCA1 in the absence or presence of irradiation were pulled down by Ni-NTA chromatography using His-tagged BRCA-Δ11 and separated by gel electrophoresis under denaturing conditions (Fig. 1a). Proteins whose binding was altered by irradiation were further analyzed by matrix-assisted laser desorption ionization-time of flight mass spectrometry. The most notable protein whose interaction with BRCA1-Δ11 increased upon irradiation in this analysis was cyclin B1. Mascot search revealed that eight mass spectrometry-predicted peptides matched to cyclin B1, covering 12% of the cyclin B1 protein sequence. Although cyclin B1 has previously been identified as a binding partner for BRCA1^15^, the significance of this interaction has remained unclear. To confirm this interaction, we expressed Myc epitope-tagged BRCA1 and FLAG epitope-tagged cyclin B1 in 293T cells and performed immunoprecipitations. As shown in Fig. 1b, BRCA1 and cyclin B1 were effectively expressed in 293T cells and shown to interact. To determine whether BRCA1 and cyclin B1 interact under physiological conditions, we tested interactions of endogenous proteins in irradiated MCF7 cells. In these experiments, cyclin B1 associated with the anti-BRCA1 antibody-precipitated complex, but not with normal IgG precipitates (Fig. 1c). Immunoprecipitation analyses of irradiated cells under overexpressed and endogenous conditions further confirmed the induction of this protein interaction by irradiation (Fig. 1d, e).

To further delineate the region(s) of BRCA1 responsible for binding cyclin B1, we generated fragments of the BRCA1-Δ11 isoform (Fig. 1f). Studies of two protein fragments spanning the length of BRCA1-Δ11 revealed that cyclin B1 interacts with the N-terminal region containing the RING domain but not with the well-known BRCT-domain-containing C-terminal fragment (Fig. 1h). Next, we sought to determine the BRCA1-binding domain in cyclin B1 by performing immunoprecipitation experiments using several truncated forms of cyclin B1 carrying a C-terminal FLAG tag (Fig. 1g). In transfected 293T cells, BRCA1-Δ11 was predominantly pulled down by the C-terminal fragment of cyclin B1 containing a cyclin box (Fig. 1i). Collectively, these results indicate that BRCA1 interacts with cyclin B1 in a DNA damage-dependent manner and that the N-terminal region of BRCA1 and C-terminal domain of cyclin B1 are required for the interaction.

### Physiological role of BRCA1–cyclin B1 interactions

**Fig. 2:**
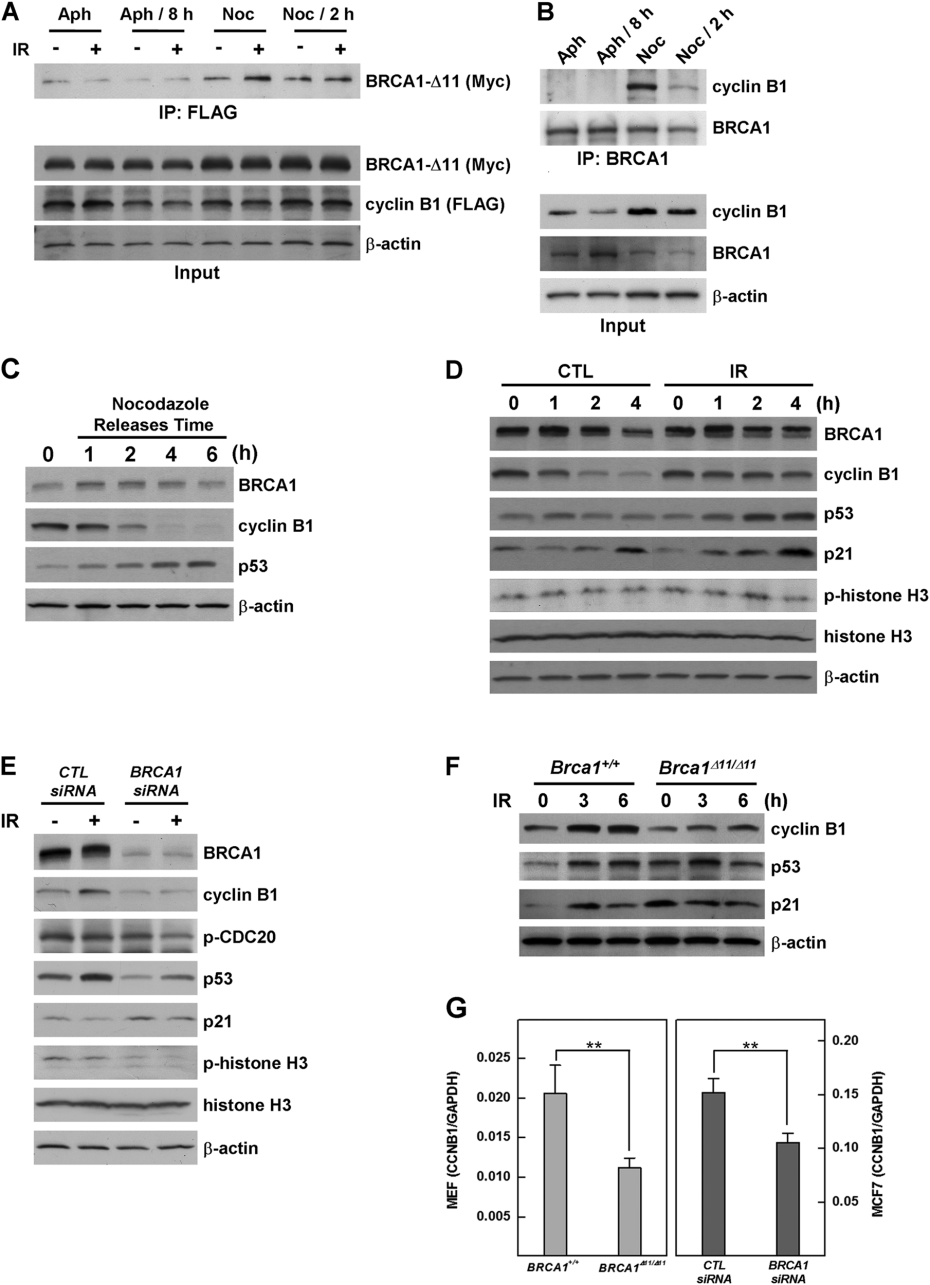
Cyclin B1 stability is dependent on cell cycle phase, DNA damage, and the presence of BRCA1. **a** MCF7 cells transfected with Myc-BRCA1-Δ11 and FLAG-cyclin B1 were treated as follows to synchronize the cell cycle: (1) aphidicolin (0.3 μM) for 24 h, (2) release for 8 h, (3) nocodazole (100 ng/mL) for 24 h, and (4) release for 2 h. After synchronization, the cells were treated without or with irradiation (10 Gy), lysed, immunoprecipitated with anti-FLAG antibody-conjugated agarose, and analyzed by Western blotting. Input represents 5% of the protein extract prior to immunoprecipitation. **b** MCF7 cells treated as above to synchronize cell cycle were lysed, and the lysates were immunoprecipitated with anti-BRCA1 IgG, followed by Western blot analyses using the indicated antibodies. **c** Protein expression patterns in MCF7 cells were analyzed after nocodazole synchronization and at various times after release. **d** MCF7 cells were synchronized by nocodazole treatment and released in the presence of irradiation (10 Gy). Protein expression patterns were analyzed at the indicated times after irradiation. **e** MCF7 cells transfected with scrambled or *BRCA1*-targeting siRNAs were synchronized by nocodazole treatment and released in the presence of irradiation for 2 h, after which protein expression patterns were analyzed by Western blotting. **f** WT (*Brca1*^*+/+*^) and *Brca1*-mutant (*Brca1*^*Δ11/Δ11*^) MEFs were irradiated, and their protein expression patterns were examined by Western blotting. **g** *CCNB1* mRNA levels were measured by quantitative RT-PCR in BRCA1-replete cells (*Brca1*^*+/+*^ MEFs and control siRNA-transfected MCF7 cells) and BRCA1-deficient cells (*Brca1*^*Δ11/Δ11*^ MEFs and *BRCA1* siRNA-transfected MCF7 cells). **P < 0.01

Like all cyclins, cyclin B1 levels oscillate over the course of the cell cycle. Immediately prior to mitosis, large amounts of cyclin B1 are present in the cell, but at the end of mitosis, cyclin B1 is targeted for degradation, permitting the cell to exit mitosis^26^. To examine the interaction of BRCA1 and cyclin B1 during the cell cycle course, we transfected MCF7 cells with both proteins and arrested the cells at different cell cycle phases, with or without radiation exposure. Cell lysates were immunoprecipitated using anti-FLAG IgG-conjugated agarose, and immunoprecipitates were analyzed by Western blotting using anti-Myc antibodies. The interaction of BRCA1 with cyclin B1 was readily detected in nocodazole-treated (M phase) cells and was increased by more than two-fold (214%) upon irradiation (Fig. 2a). To confirm the cell cycle-dependent interaction of BRCA1 and cyclin B1 under physiological conditions, we tested interactions of endogenous proteins during various cell cycle phases. As shown in Fig. 2b, the interaction of BRCA1 and cyclin B1 was strongest in mitotic arrest induced by nocodazole treatment and gradually decreased during release from the synchronization, whereas interactions were not readily detected in the aphidicolin-arrested and aphidicolin-released cells. Interestingly, the interaction pattern of BRCA1 and cyclin B1 corresponded with the level of cyclin B1, suggesting their interaction is involved in the stabilization of cyclin B1.

To assess the levels of BRCA1 and cyclin B1 during mitosis progression, we synchronized MCF7 cells at early M phase via treatment with nocodazole and analyzed their protein expression patterns at the indicated times after release. As shown in Fig. 2c, BRCA1 levels increased immediately after release from nocodazole treatment and subsequently decreased, whereas cyclin B1 levels decreased dramatically, indicating exit from mitosis. Interestingly, when mitosis progression was restarted, irradiation dramatically increased the level and phosphorylation status of BRCA1 and attenuated cyclin B1 degradation (Fig. 2d), suggesting that DNA damage during mitosis inhibits the degradation of cyclin B1, preventing cell cycle progression.

Next, we tested whether BRCA1 contributes to the stability of cyclin B1 during mitosis progression in the context of DNA damage. Indeed, cyclin B1 was destabilized in irradiated MCF7 cells in which BRCA1 expression was knocked down by *BRCA1*-targeting siRNA (Fig. 2e). In addition, the cyclin B1 level in *Brca1*-mutant (*Brca1*^*Δ11/Δ11*^) MEFs exposed to radiation was less than half (48%) than that in irradiated WT (*Brca1*^*+/+*^) MEFs (Fig. 2f). Taken together, these data demonstrate that BRCA1 is required for maintenance of cyclin B1 protein stability in the context of DNA damage.

**Fig. 3:**
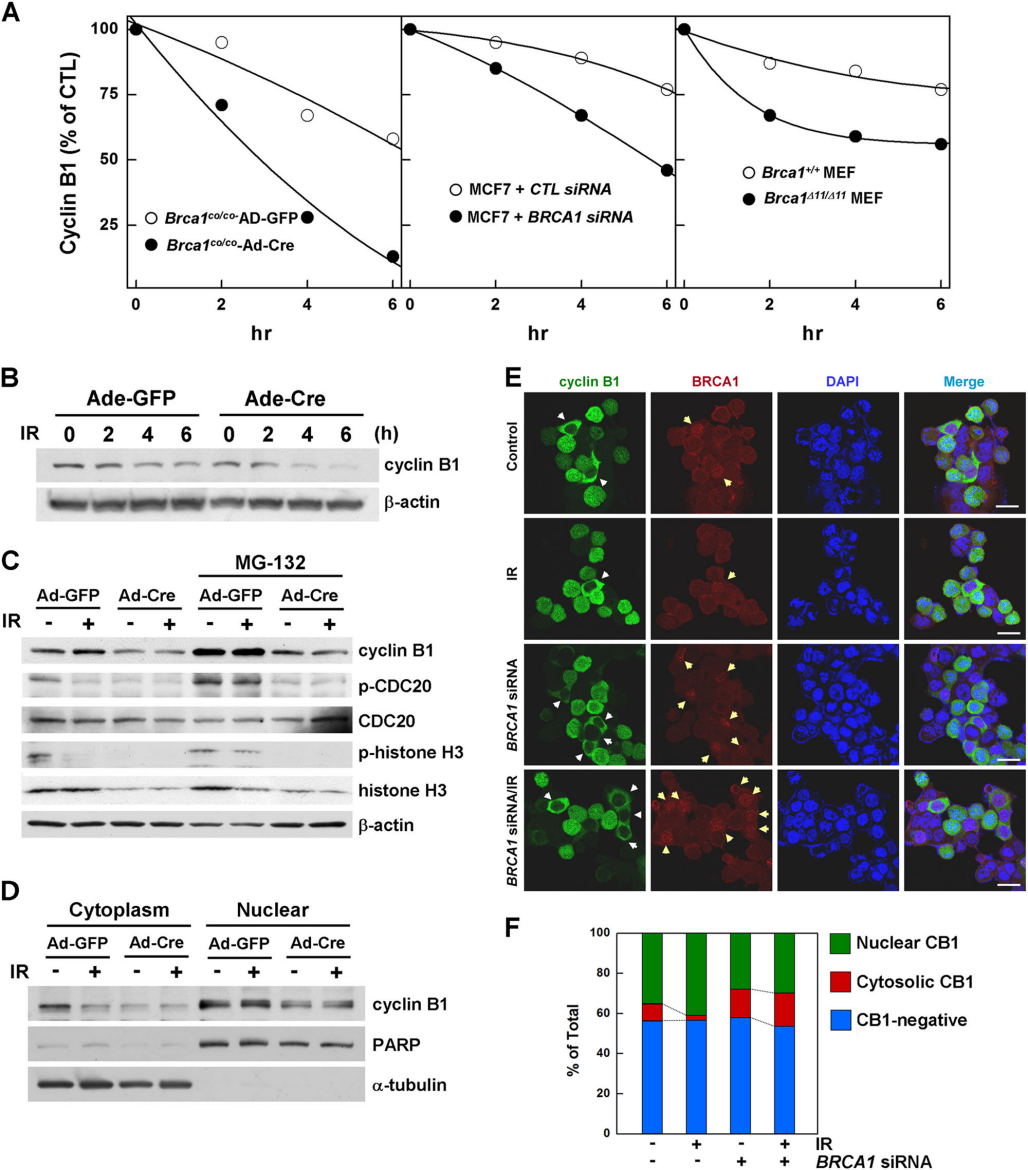
BRCA1 is involved in the stability and translocation of cyclin B1. **a** Levels of cyclin B1 in BRCA1-replete cells (*Brca1*^*co/co*^ MEFs infected with Ad-GFP, MCF7 cells infected with control siRNA, and *Brca1*^*+/+*^ MEFs) and BRCA1-deficient cells (*Brca1*^*co/co*^ MEFs infected with Ad-Cre, MCF7 cells infected with *BRCA1* siRNA, and *Brca1*^*Δ11/Δ11*^ MEFs) in the context of mitotic DNA damage. Cells were synchronized by nocodazole treatment and released with exposure to radiation (10 Gy) in the presence of cycloheximide (100 ng/mL), which was included to prevent further protein synthesis. Levels of cyclin B1 at the indicated times were analyzed by Western blotting; β-actin was used as the loading control. **b** MEFs from *Brca1* conditional-knockout embryos (*Brca1*^*co/co*^) were infected with adenovirus-expressing (Ad-Cre) to delete *Brca1* or with empty adenovirus (Ad-GFP) as a control. Levels of cyclin B1 were examined by Western blotting; β-actin was used as the loading control. **c** Loss of BRCA1 resulted in failure to stabilize cyclin B1 protein upon irradiation. *Brca1*^*co/co*^ MEFs infected with Ad-Cre or Ad-GFP were synchronized and released with irradiation (10 Gy) in the absence or presence of MG132 (10 μM). Protein expression patterns were examined by Western blot analysis. **d** *Brca1* is required for DNA damage-induced nuclear localization of cyclin B1. *Brca1*^*co/co*^ MEFs infected with Ad-Cre or Ad-GFP were synchronized and released, with or without irradiation, in the presence of MG132 (10 μM). Nuclear and cytoplasmic fractions were subsequently separated and analyzed by Western blotting. PARP and α-tubulin were used as controls for nuclear and cytoplasmic fractions, respectively. **e** Scramble-transfected or *BRCA1* siRNA-transfected MCF7 cells were synchronized by nocodazole treatment and released in the absence or presence of radiation. The cells were stained with anti-cyclin B1 antibody (green), anti-BRCA1 antibody (red), and DAPI (blue). White (left panels) and yellow (second left panels) arrows indicate cells with cyclin B1 in the cytosol and BRCA1 foci in the nuclei, respectively. Scale bars in merged images: 10 μm. **f** Percentages of cells depending on the cyclin B1 status are shown. At least 500 MCF7 cells in eight different fields were counted in each staining sample for calculation of cyclin B1 localization

Reverse transcription-PCR (RT-PCR) estimates showed that depletion of BRCA1 resulted in a 46 and 30% reduction in cyclin B1 messenger RNA (mRNA) levels in MEFs and MCF7 cells, respectively (Fig. 2g). Thus, although cyclin B1 mRNA was downregulated in BRCA1-deficient cells, the dramatic reduction in cyclin B1 protein in irradiated mitotic cells suggests a contribution of post-translational regulation. To test this possibility, we examined the levels of cyclin B1 in BRCA1-replete and BRCA1-deficient mitotic cells exposed to radiation. In these experiments, BRCA1 was deleted by infecting *Brca1* conditional MEFs with an Ad-Cre, after which the cells were synchronized by nocodazole treatment, released in the presence of cycloheximide (100 ng/mL) to inhibit further protein synthesis, and then irradiated with 10 Gy. The levels of cyclin B1 at 6 h after irradiation were 58 and 13% in cells infected with control adenovirus and Cre-recombinase-containing adenovirus, respectively (Fig. 3a (left panel) and 3b). Additionally, cyclin B1 degradation in *BRCA1*-knockdown MCF7 cells and *Brca1*^*Δ11/Δ11*^ MEFs was 2.3 and 2.0 times more rapid, respectively, compared with control cells (Fig. 3a, middle and right panels). Moreover, preventing cyclin B1 degradation by treating cells with irradiation resulted in an increase in cyclin B1 levels, but the level of cyclin B1 was unchanged by deletion of BRCA1, indicating irradiation-induced cyclin B1 stabilization requires BRCA1 (Fig. 3c).

One important mechanism by which cyclin B1-Cdk1 activity is regulated is through regulation of its subcellular localization. Upon initiation of mitosis, cyclin B1 is translocated to the nucleus, where it becomes stabilized; it is subsequently exported to the cytoplasm, permitting its degradation and exit from mitosis^27^. To test the role of subcellular localization in cyclin B1 stability, we first infected *Brca1* conditional-knockout MEFs with Ad-Cre to delete *Brca1* or with empty adenovirus (Ad-GFP; control) and synchronized cells in mitosis through treatment with nocodazole. We then released cells from the nocodazole block, exposed them to irradiation in the presence of the proteasome inhibitor MG132, and examined the cyclin B1 level in nuclear and cytoplasmic fractions of cell extracts. WT MEFs showed a decrease in cytoplasmic cyclin B1 after irradiation, whereas cyclin B1 was not reduced in the cytoplasm under the same conditions in BRCA1-deleted cells (Fig. 3d).

To assess whether subcellular cyclin B1 localization is determined by BRCA1 and DNA damage, we examined the localization of cyclin B1 and BRCA1 in *BRCA1* siRNA-transfected and/or siRNA-irradiated MCF7 cells. As shown in Fig. 3e, we found that the MCF7 cells with nuclear cyclin B1 also exhibited BRCA1 in the nucleus, with dispersed localization, indicating that nuclear cyclin B1 overlapped with BRCA1 localization. On the other hand, most of the cells with cytosolic cyclin B1 displayed punctate BRCA1 in the nucleus, which is frequently detected with DNA damage^28^. These results suggest that BRCA1 is co-localized with cyclin B1 and its downregulation increased the number of cells with a BRCA1-associated DNA damage response and cytosolic translocation of cyclin B1. In addition, the portion of MCF7 cells with cytosolic cyclin B1 was higher in *BRCA1* siRNA-transfected MCF7 cells (14.2%) than in scramble siRNA-transfected cells (8.3%, Fig. 3f). Taken together, these results indicate that BRCA1 prevents cyclin B1 proteolysis in DNA-damaged mitotic cells by regulating the subcellular localization of cyclin B1.

### Rescue of cyclin B1 in BRCA1-deficient cells reduces survival

**Fig. 4:**
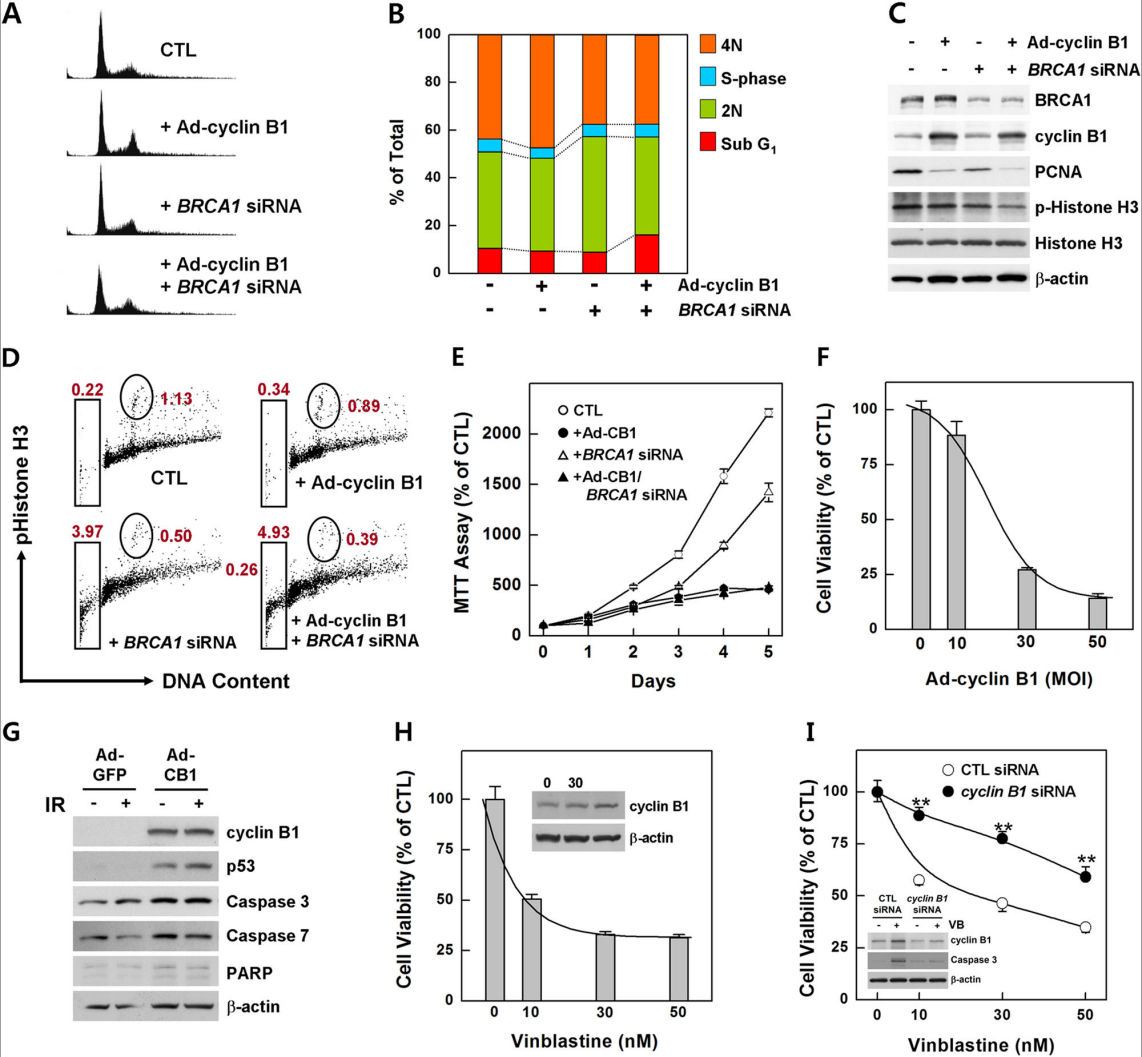
Overexpression of cyclin B1 reduces the survival of BRCA1-deficient cells. **a** Unsynchronized MCF7 cells in which BRCA1 was knocked down and/or cyclin B1 was overexpressed were examined according to the cell cycle phase, determined by flow cytometry. Representative histograms showing the DNA content in MCF7 cells with BRCA1 depletion and/or cyclin B1 overexpression. **b** Percentages of cells in each cell cycle phase are shown. **c** Protein expression patterns in *BRCA1*-knockdown and/or cyclin B1-overexpressing MCF7 cells were analyzed via Western blot analysis. **d** To count the cells in G2/M transition, unsynchronized MCF7 cells transfected with BRCA1 siRNA and/or transduced with Ad-cyclin B1 were stained with phospho (Ser10)-Histone H3 and analyzed via flow cytometry. **e** Survival of cyclin B1-overexpressing MCF7 cells was measured in cells transfected with scrambled siRNA or *BRCA1*-targeted siRNA. The results are expressed as survival relative to that at infection or transfection. **f** A breast cancer cell line derived from a *Brca1*^*Δ11/Δ11*^*p53*^*−/−*^ mouse was infected with increasing amounts of Ad-cyclin B1, after which survival was analyzed with an MTT assay. **g** A breast cancer cell line derived from a *Brca1*^*Δ11/Δ11*^*p53*^*−/−*^ mouse was infected with Ad-cyclin B1 or Ad-GFP (control), after which the protein pattern was analyzed via Western blotting. **h** Estimated survival of *Brca1*^*Δ11/Δ11*^*;p53*^*−/−*^ mammary tumor cell lines at the indicated concentration of vinblastine. **i** The survival of *Brca1*^*Δ11/Δ11*^*p53*^−/−^ mammary tumor cell lines was assessed after transfection with scramble or *Ccnb1* siRNA and treatment with vinblastine at the indicated concentration. **P < 0.01

We previously examined the levels of cyclins in several cell lines generated from tumors from *Brca1*-mutant mice. Compared with normal mammary tissues, cyclin D1 was overexpressed in asynchronous cultured cells from *Brca1*-mutant tumors, but cyclins A, B1, and E were not detectable, indicating that mammary tumors caused by the loss of BRCA1 are associated with a deficiency of cyclin B1^16^. To assess the relationship between cyclin B1 and BRCA1 deficiency, we restored cyclin B1 in MCF7 cells by infection with an Ad-cyclin B1, followed by knockdown of the *BRCA1* gene. We then determined the cell cycle distribution in *BRCA1*-knockdown and/or cyclin B1-expressing MCF cells via FACS analysis. Unexpectedly, our FACS analysis of DNA content revealed that cyclin B1-overexpressing/*BRCA1*-knockdown MCF7 cells exhibited a dramatically increased apoptotic sub-G_1_ population (16.1%) compared with control (10.4%), cyclin B1-overexpressing (9.3%), and *BRCA1*-knockdown cells (8.8%) (Fig. 4a, b). Further Western blot analyses showed a reduction in PCNA in cyclin B1-overexpressing/*BRCA1*-knockdown MCF7 cells, indicating that overexpression of cyclin B1 attenuated the proliferation of BRCA1-deficient cells (Fig. 4c). We then determined the G2/M transition in *BRCA1*-knockdown and/or cyclin B1-expressing MCF7 cells by measuring phospho (Ser10)-histone H3. As shown in Fig. 4d, the ratio of transiting G2/M checkpoint cells was reduced in Ad-cyclin B1-infected and *BRCA1* siRNA-transfected cells (0.39%) compared with control (1.13%), Ad-cyclin B1-infected (0.89%) and *BRCA1*-knockdown (0.50%) cells, suggesting induction of cyclin B1 in BRCA1-deficient cells altered the rate of G2/M transition. Importantly, the apoptotic sub-G1 population appeared higher in cyclin B1-overexpressing and *BRCA1*-knockdown cells (4.93%) compared with control (0.22%), cyclin B1-overexpressing (3.97%), and *BRCA1*-knockdown (3.97%) cells. Taken together, these results suggested that the proliferation and apoptosis of mitotic cells were affected by the alterations of BRCA1 and cyclin B1. Next, to determine whether induction of cyclin B1 in BRCA1-deficient cells influences cell survival, we assessed cell viability using MTT assays. After 5 days, the number of MCF7 cells increased 14-fold in the absence of BRCA1, whereas that of cyclin B1-overexpressing BRCA1-deficient MCF7 cells only increased 4.8-fold over the same period (Fig. 4e). These results suggest that induction of cyclin B1 efficiently attenuates the proliferation of BRCA1-deficient cells in association with a reduction in the mitotic cell population and an increase in the number of apoptotic cells.

We next tested whether overexpression of cyclin B1 attenuates the survival of BRCA1-associated tumor cells. A breast cancer cell line derived from a *Brca1*^*Δ11/Δ11*^*;p53*^*−*^^*/−*^ mouse tumor was infected with increasing doses of Ad-cyclin B1, and cell survival was measured with MTT assays. As shown in Fig. 4f, the proliferation of *Brca1*-mutant tumor cells decreased as the amount of cyclin B1 increased. In addition, overexpression of cyclin B1 in *Brca1*-mutant tumor cells increased the levels of apoptosis-promoting proteins, including p53, caspase-3, caspase-7, and PARP (Fig. 4g), indicating that overexpression of cyclin B1 in BRCA1-mutant tumor cells reduces cell survival in association with induction of apoptosis.

Vinblastine, a microtubule inhibitor, targets tubulin or microtubules and suppresses microtubule dynamic instability, leading to prolonged activation of the spindle checkpoint and mitotic arrest^29^. It was recently shown that vinblastine causes a sustained increase in endogenous levels of cyclin B1^30^. To examine the effect of vinblastine on BRCA1-associated breast cancer, we assessed the survival of *Brca1*^*Δ11/Δ11*^*p*53^−/−^mammary tumor cell lines following treatment with increasing concentrations of vinblastine. These experiments showed that survival of *Brca1*-mutant tumor cells decreased with each increment in cyclin B1 induced by successively higher concentrations of vinblastine (Fig. 4h). To further investigate the contribution of cyclin B1 in the vinblastine-induced growth suppression of BRCA1-associated breast cancer cells, we transfected the *Brca1*^*Δ11/Δ11*^*p53*^−/−^ mammary tumor cell line with siRNA against *Ccnb1* and examined the effect of vinblastine. As shown in Fig. 4i, the growth suppression induced by vinblastine was significantly reduced in *Ccnb1* siRNA-transfected cells (*P* < 0.01). The level of cleaved caspase-3 was also decreased in vinblastine-treated *Ccnb1* siRNA-transfected cells compared with scramble siRNA-transfected cells.

### Vinblastine treatment attenuates tumor growth in mice

**Fig. 5:**
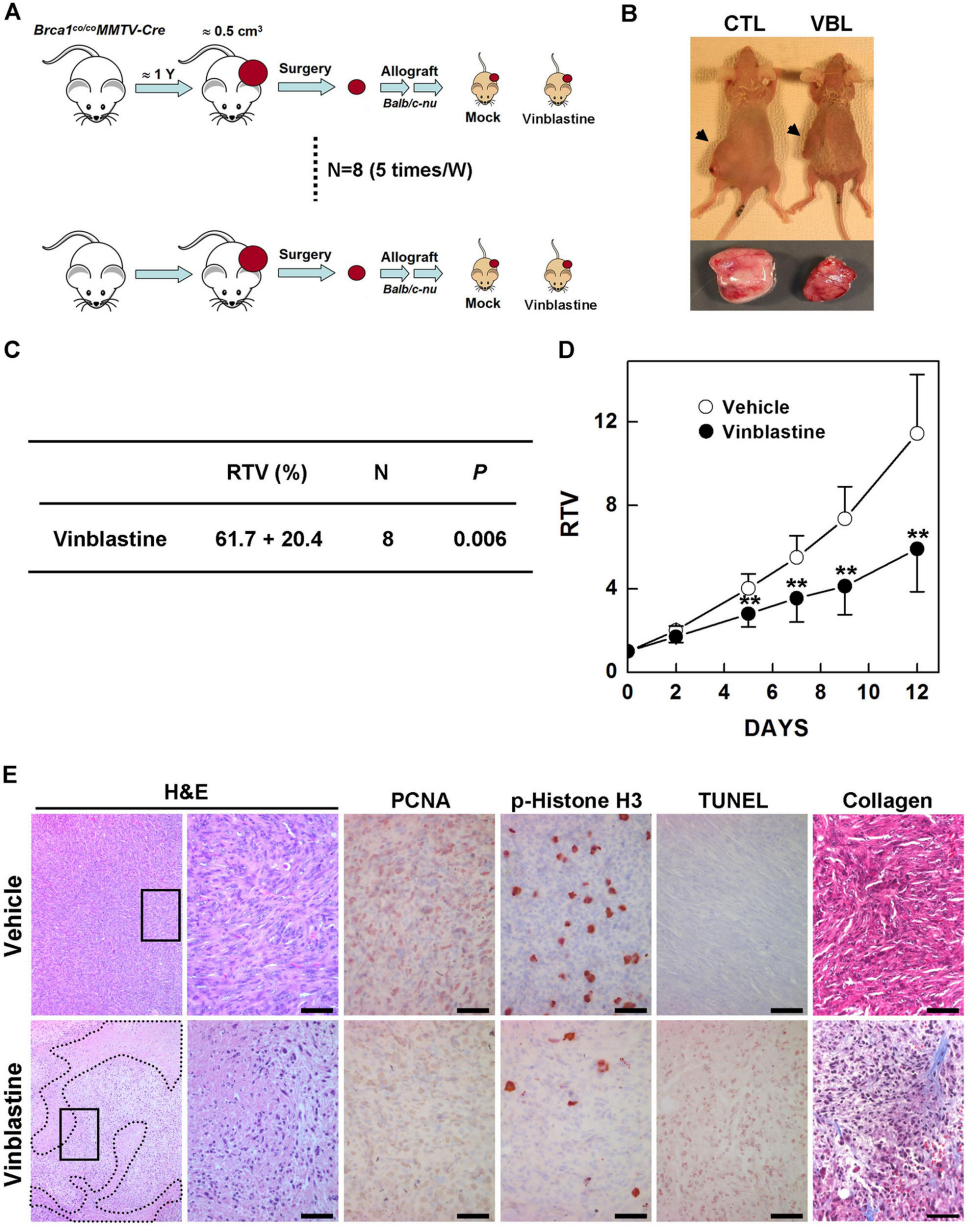
Therapeutic effects of vinblastine in a *Brca1*-deficient tumor transplantation model. **a**, **b** Overview of the allograft model and drug treatments. Eight spontaneously developed mammary tumors were collected from *Brca1*^*co/co*^*MMTV-Cre* mice and transplanted into nude mice. Tumor growth was tested following treatment with vehicle or vinblastine (VBL; 0.5 mg/kg, IP, 5 times/week). After the tumor of any mouse implanted with the same original tumor reached ~3000 mm^3^, all the mice implanted with that tumor were sacrificed and examined. **c** Summary of the allograft experiment. The values represent the means ± SD. **d** Responses of allotransplanted *Brca1*-mutant mammary tumors to vinblastine. **P < 0.01. **e** Histological analysis of tumors from vehicle-treated and vinblastine-treated mice. Areas highlighted by dotted lines represent the stromal region. In hematoxylin and eosin (H&E) staining images, the panels at the right are a magnification of the boxed areas in the adjacent panels. Scale bars: 50 μm

Previous studies have reported that mammary tumors that spontaneously develop in BRCA1-deficient mice can be orthotopically transplanted into female mice without loss of their original phenotype, gene expression profile, or sensitivity to anticancer agents^31,32^. To test whether the mitotic arrest associated with induction of cyclin B1 suppresses *BRCA1*-mutant breast cancer in vivo, we examined the effect of vinblastine in an allograft model of transplanted mammary tumor tissues from *Brca1*^*co/co*^*MMTV-cre* mice, which spontaneously develop mammary tumors starting at ~10 months of age. We collected tumor tissues from eight spontaneously developed mammary tumors from *Brca1*^*co/co*^*MMTV-cre* mice and orthotopically transplanted them into HsdCpb:NMRI-*Foxn1*^*nu*^ (nude) female mice. We then used this model to test the efficacy of vinblastine by comparing treated and non-treated tumors of the same tumor origin (Fig. 5a, b). The overall RTV (comparing treated and non-treated tumors) for allograft mice bearing *Brca1*-mutant tumors treated with vinblastine was 61.7% (Fig. 5c). A comparison of baseline and progressed tumor volumes showed that vinblastine caused a significant reduction in volume from day 5 after treatment until the tumor volume reached ~3000 mm^3^ (Fig. 5d). At 2 weeks after initiation of the treatment, the volume of tumors from control mice had increased 11.4-fold compared with baseline, whereas the volume of tumors from vinblastine-treated mice was significantly smaller (5.9-fold increase). In addition, vinblastine-treated tumors exhibited large stromal areas in association with a reduction in proliferation markers, including PCNA and phospho-histone H3, accumulation of collagen fibrosis, and higher levels of TUNEL staining compared with non-treated tumor tissues (Fig. 5e). These findings suggest that administration of vinblastine attenuates the proliferation and stimulates the death of *Brca1*-associated tumors.

### Vinblastine response-associated genes

**Fig. 6:**
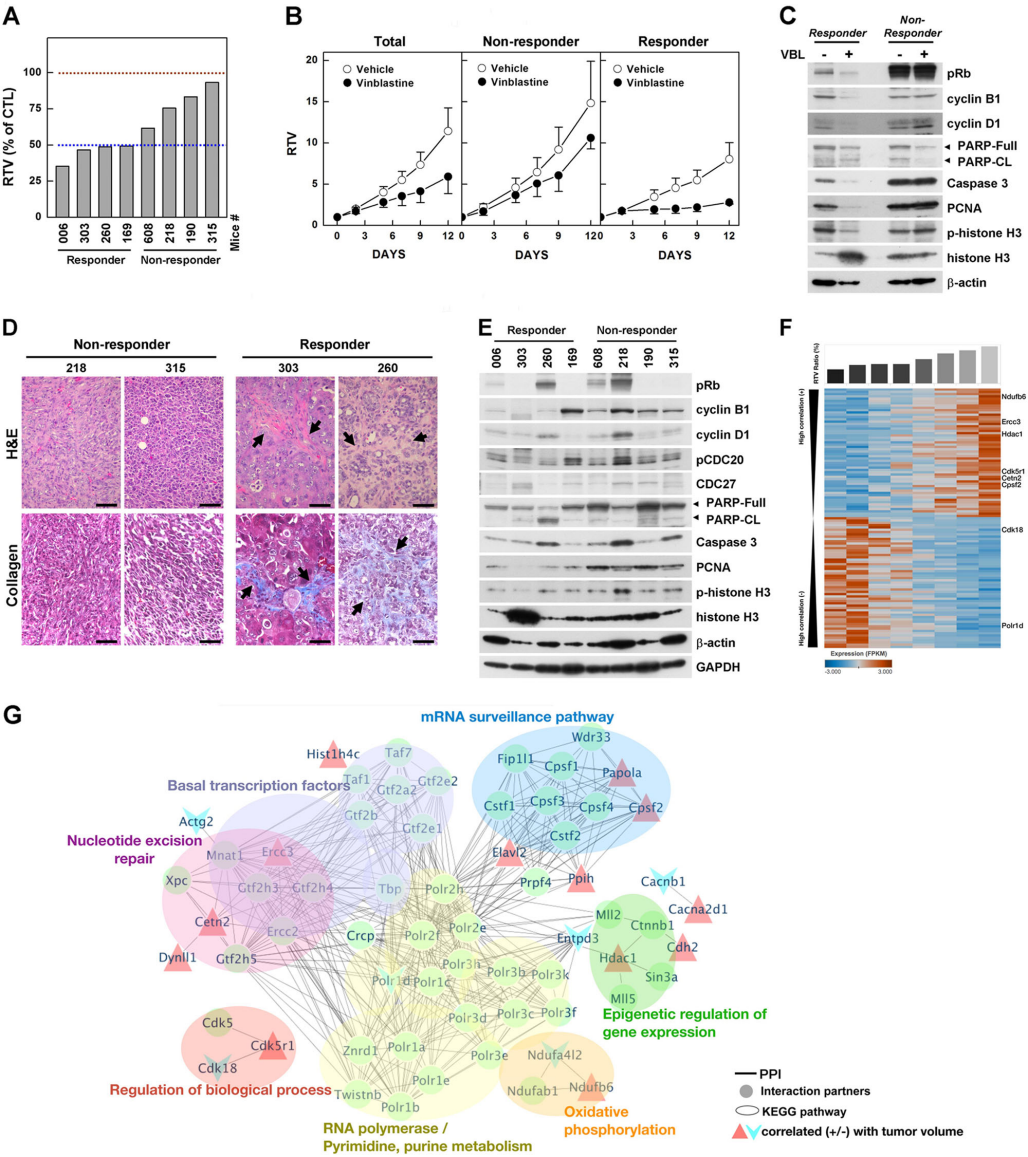
Analysis of vinblastine response-associated biomarkers. **a** Graph showing calculated RTVs (RTV of treated tumor/RTV of control tumor × 100) for individual tumors from mice treated with vinblastine. **b** Responses of BRCA1-allograft tumors to vinblastine, segregated based on the RTV (non-responder, RTV >50; responder, RTV <50). **c** Protein expression patterns in responder and non-responder tumors from vehicle-treated and vinblastine (VBL)-treated mice. Histone H3 and β-actin were used as loading controls. **d** Histological analyses of vinblastine-treated tumors from responders and non-responders are shown. The arrows indicate the stroma (upper panels) and fibrosis with collagen deposition (blue; lower panels). Scale bars: 50 μm. **e** Protein expression patterns in baseline tumors from responders and non-responders. Histone H3, β-actin, and GAPDH were used as loading controls. **f** Heat map showing downregulation and upregulation of selected genes in the vinblastine-responder group compared with the non-responder group in the allograft model; *P* < 0.01. Tumor samples are sorted with respect to their RTV to highlight correlations with gene expression. **g** Integrated functional network analysis of the selected genes using the STRING protein interaction network and KEGG pathways

Although vinblastine treatment significantly improved the therapeutic outcome in *Brca1*-mutant tumors, the responses to this mitosis-targeting and cyclin B1-targeting strategy were not uniform. Indeed, an analysis of tumors in individual mice treated with vinblastine revealed that four of the eight mice exhibited a more than 50% reduction in tumor volume in response to vinblastine treatment, whereas the remainder showed a <50% reduction (Fig. 6a). Stratification of vinblastine-treated mice according to their RTVs (responders, RTV < 50%; non-responders, >50%), non-responder mice (*n* = 4) showed a 29.6% reduction in RTV, whereas responders (*n* = 4) exhibited a 65.3% decrease in RTV (Fig. 6b). A further analysis of tumor proteins revealed that vinblastine-treated tumors from responders showed a decrease in proliferation markers and an increase in apoptotic markers, whereas non-responders showed no major change in markers after vinblastine treatment (Fig. 6c). In addition, tumors from responders displayed enlarged stromal areas and fibrosis after vinblastine treatment, whereas non-responders showed no histological alterations after the same treatment (Fig. 6d).

Maximizing the therapeutic benefit of vinblastine treatment of BRCA1-associated mammary tumors would be facilitated by the ability to distinguish potential responders by analyzing the tumor at baseline (i.e., before initiation of treatment). In an effort to identify prognostic markers of potential responder candidates before treatment, we classified the cases based on their responsiveness to vinblastine treatment and analyzed the protein patterns in untreated tumor tissue. As shown in Fig. 6e, Western blot analyses showed that the levels of cyclin B1 and PCNA were frequently decreased in the responder group compared with the non-responder group, indicating that PCNA/cyclin B1-low BRCA1-associated tumors are suitable candidates for vinblastine treatment.

In addition, to increase the potential clinical efficacy of vinblastine against BRCA1-deficient breast cancer, we also sought to identify specific efficacy-associated genes by examining gene expression patterns in untreated tumors. To predict which genes would show an expression pattern that correlated with RTV following vinblastine treatment of *Brca1*-deleted tumors, we screened the entire transcriptome using mRNA sequencing data and the Cufflinks computational pipeline. Using this approach, we identified 113 HCGs (significance level <0.01; Spearman’s rank correlation >0.7) (Fig. 6f and Supplementary Table 1). Gene ontology (GO) analyses revealed that 59 of the 113 (52%) HCGs were involved in RNA metabolic process (GO:0016070), 61 (52%) were involved in gene expression (GO:0010467), and the remainder were involved in cell organization, biogenesis, biosynthesis, and morphogenesis (Supplementary Figure 1 and Supplementary Table 2).

To assess the downstream effects of the 113 putative marker genes following vinblastine treatment, we identified downstream pathways that might specifically affect the regulation of vinblastine resistance or sensitivity. To this end, we constructed a protein–protein interaction (PPI) network of the 113 genes and their interacting molecules using the STRING database^24,25^ and KEGG pathways. This analysis showed that the 113 genes were connected to various pathways through a previously annotated PPI in the constructed network (Fig. 6g and Supplementary Table 3). It also revealed seven clusters of proteins connected to various biological functions and cellular components (false discovery rate <0.05). Among the seven clusters, one was connected to the function “nucleotide excision repair”. DNA-damaging chemotherapeutic drugs also evoke mechanisms that allow cells to repair damage and confer resistance to anticancer drugs^33^. In this module, Ercc3 and Cetn2 (centrin 2) were positively correlated with tumor size. Other modules, including “epigenetic regulation of gene expression” and “mRNA surveillance pathway,” might also be associated with vinblastine resistance. Additionally, the network facilitated identification of the RNA polymerase subunits Polr1d and Polr2e/f/h, as well as Tbp (TATA-box binding protein), as potentially being responsible for the crosstalk between “nucleotide excision repair” and other categories.

## Discussion

BRCA1-deficient breast cancer is an extensively studied hereditary cancer that exhibits significantly higher levels of chromosomal abnormality than sporadic breast cancers^16,34,35^. Loss of BRCA1 is associated with deregulation of cell cycle checkpoints, especially G2/M and spindle checkpoints. Because of these defects, *Brca1-*mutant cells accumulate DNA damage owing to chromosome missegregation and genetic instability^17,36^.

Herein, we show that BRCA1 interacts with cyclin B1 in a manner that depends on cell cycle and DNA damage, and its loss decreases the stability of cyclin B1. A previous immunoprecipitation-based study showed that BRCA1 interacts with several CDKs/cyclins, including cyclin B1, suggesting a contribution of BRCA1 to regulation of cell cycle and proliferation^15^. In the current study, we demonstrated that the interaction of BRCA1 with cyclin B1 was dramatically increased by irradiation-induced double-strand DNA breaks. In addition, these proteins interacted to a greater extent in the mitotic phase, during which cyclin B1 levels are the highest^37^. We then investigated whether the loss of BRCA1 influenced the stability of cyclin B1 during mitosis in the context of DNA damage. Interestingly, during mitosis, cyclin B1 was dramatically stabilized upon irradiation in the presence of BRCA1 but was not protected from degradation in the absence of BRCA1. Further analysis revealed that BRCA1 is responsible for regulating cyclin B1 not only at the transcriptional level but also through post-translational modification, indicating that BRCA1 is required for the maintenance of cyclin B1. In contrast, previous reports have suggested that cyclin B1 is degraded independent of APC/C by BRCA1 in a ubiquitin-dependent manner, and further reports have indicated that cyclin B1 is a bona fide substrate of BRCA1 E3 ligase^19,38^.

Two possibilities might explain the discrepancies surrounding the contribution of BRCA1 to cyclin B1 stability. First, the level of cyclin B1 is precisely regulated during cell cycle progression and is readily detected from the initiation of mitosis to exit from mitosis. Therefore, when cells are damaged during mitosis, BRCA1 is able to protect cyclin B1 from degradation and thereby prevent exit from mitosis, causing cell cycle arrest in the middle of mitosis and attenuating the proliferation of damaged cells. Thus, the contribution of BRCA1 to cyclin B1 function during mitosis in the context of DNA damage is quite different from that under asynchronous conditions, in which overall cyclin B1 levels are lower compared with those in mitosis. The second possibility reflects the activity of BRCA1 as a double-edged sword. MEFs carrying a targeted deletion of the *Brca1* gene exhibit apoptosis in association with extensive chromosomal abnormalities^36^. However, overexpression of BRCA1 has also been shown to induce an apoptotic signaling pathway involving c-Jun N-terminal kinase^39,40^. Interestingly, loss of BRCA1 leads to a defective G2/M checkpoint in association with unequal chromosome segregation, abnormal nuclear division, and aneuploidy. However, induction of BRCA1 also results in bizarre abnormalities at the G_2_/M boundary, indicating that an alteration in BRCA1 in either direction—deficiency or excess—is able to cause similar physiological events, including chromosomal abnormalities, mitotic defects, and apoptosis.

Notably, the most striking phenotype in this study was induction of cyclin B1 in BRCA1-deficient cells, which led to synthetic lethality. Considering this strategy, we also provided evidence that modulating mitotic progression and cyclin B1 expression could be therapeutically beneficial in the treatment of BRCA1-associated breast cancer. In addition to the results obtained in our in vitro studies of cyclin B1 overexpression using adenovirus-expressing cyclin B1, we found that vinblastine treatment of an allograft tumor model significantly reduced tumor progression. After 2 weeks, the volume of tumors in mice treated with vehicle increased 11.4-fold compared with baseline, whereas vinblastine-treated tumors were significantly smaller (5.9-fold relative to baseline), with enlarged stromal fibrosis, reduced expression of proliferation markers, and induction of apoptosis.

Importantly, our in vivo results showed that tumor-bearing mice harboring the same *Brca1* mutation (*Brca1-*Δ*11*) exhibited non-uniform responses to vinblastine. Although vinblastine treatment generally triggered significant responses in preclinical trials using an allograft model, tumor progression in some mice resembled that of the corresponding untreated control mice. In our allograft study, tumors in four of the eight mice treated with vinblastine were more than 50% smaller than those of their control counterparts, whereas tumors in the remaining four mice showed a lesser response. When vinblastine-treated mice were classified according to their status as responders (RTV < 50%) or non-responders (RTV > 50%), vinblastine non-responders showed a 29.6% reduction in RTV (vs. 14.8 and 10.6 times for vehicle and vinblastine treatment, respectively), which was much smaller than the 65.3% decrease in RTV observed for responders (vs. 8.0 and 2.8 times for vehicle and vinblastine treatment, respectively). Our biochemical and histological analyses showed that responders displayed other responses to vinblastine that were different from those of non-responders. Western blot analyses revealed that vinblastine decreased the expression of proliferation markers and increased that of apoptotic markers in responders; by comparison, these markers were largely unchanged after vinblastine treatment in non-responders. In addition, vinblastine-induced enlargement of stromal areas and fibrosis was detected in the responder cohort, suggesting that tumors were damaged and scarred only in responders.

The ability to distinguish vinblastine responders from non-responders would be helpful for maximizing the therapeutic benefit of vinblastine therapy in BRCA1-associated breast cancer. Our Western blot analysis of tumors at baseline showed that reductions in cyclin B1 and PCNA were frequently identified in the responder group relative to the non-responder group. In addition, whole-transcriptome screens of baseline tumors identified 113 genes that were highly correlated with responsiveness to vinblastine; these genes encoded proteins involved in RNA metabolic processes, gene expression, cell organization, biogenesis, biosynthesis, and morphogenesis. A further analysis of PPI networks of these genes revealed that a number of protein clusters, including nucleotide excision repair, epigenetic regulation, and the mRNA surveillance pathway, were associated with vinblastine resistance.

Considering the current limitations in available treatment options for BRCA1-associated breast cancer, preclinical simulation in a mouse model, such as our approach using *Brca1*-mutant mice bearing endogenous tumors, will remain a useful strategy for testing treatment efficacy. The results obtained in the current study using this mouse simulation system suggest that targeting of mitosis and cyclin B1 could be a useful strategy for treating BRCA1-associated breast cancer. Additional studies will be required to realize the potential of this strategy and to confirm the utility of these markers for future clinical application with vinblastine treatment of BRCA1-associated breast cancer.

## Electronic supplementary material

Supplementary Figures and Tables
