# Supplementary material for: Cyclin B1 stability is increased by interaction with BRCA1, and its overexpression suppresses the progression of BRCA1-associated mammary tumors
Source: Exp Mol Med. 2018 Oct 16;50(10):136. doi: 10.1038/s12276-018-0169-z (PMC6191436; doi:10.1038/s12276-018-0169-z)
Supplement: Supplementary file 1 — Supplementary Figures and Tables [file 12276_2018_169_MOESM1_ESM.pdf]

Supplemental Information

Cyclin B1 stability is increased by interaction with BRCA1 and its overexpression suppresses the progression of BRCA1-associated mammary tumors

Eun Kyung Choi, Jeong-A Lim, Jong Kwang Kim, Moon Sun Jang, Sun Eui Kim, Hye Jung Baek, Eun Jung Park, Tae Hyun Kim, Chu-Xia Deng, Rui-Hong Wang, and, Sang Soo Kim

Supplementary Table 1. List of vinblastine response-associated genes

Symbol	Name	Correlation with RTV	Feature Type	Chr	Strand	Start	End	Ensembl ID	Entrez Gene ID
6720483E21Rik	RIKEN cDNA 6720483E21 gene	0.976190476190476	lincRNA gene	1	-	20888650	20890473	ENSMUSG00000097934	77741
Ccdc66	coiled-coil domain containing 66	0.976190476190476	protein coding gene	14	-	27481090	27508460	ENSMUSG00000046753	320234
Gtf2a1	general transcription factor II A, 1	0.976190476190476	protein coding gene	12	-	91555916	91590487	ENSMUSG00000020962	83602
Crhbp	corticotropin releasing hormone binding protein	0.975900072948533	protein coding gene	13	-	95431376	95444831	ENSMUSG00000021680	12919
Ndufb6	NADH dehydrogenase (ubiquinone) 1 beta subcomplex, 6	0.952380952380952	protein coding gene	4	-	40270591	40279421	ENSMUSG00000071014	230075
Ppih	peptidyl prolyl isomerase H	0.952380952380952	protein coding gene	4	-	119300010	119320546	ENSMUSG00000060288	66101
Ppp4c	protein phosphatase 4, catalytic subunit	0.952380952380952	protein coding gene	7	-	126785866	126792496	ENSMUSG00000030697	56420
Baat	bile acid-Coenzyme A: amino acid N-acyltransferase	0.951876318105963	protein coding gene	4	-	49489422	49506557	ENSMUSG00000039653	12012
Dmrt2	doublesex and mab-3 related transcription factor 2	0.946124746911475	protein coding gene	19	+	25672420	25679010	ENSMUSG00000048138	226049
Aipl1	aryl hydrocarbon receptor-interacting protein-like 1	0.934148484292342	protein coding gene	11	-	72027963	72037509	ENSMUSG00000040554	114230
9530077C05Rik	RIKEN cDNA 9530077C05 gene	0.928571428571428	protein coding gene	9	+	22411513	22444681	ENSMUSG00000036411	68283
Ccdc124	coiled-coil domain containing 124	0.928571428571428	protein coding gene	8	-	70868227	70873935	ENSMUSG00000007721	234388
Cdh2	cadherin 2	0.928571428571428	protein coding gene	18	-	16588877	16809246	ENSMUSG00000024304	12558
Dynll1	dynein light chain LC8-type 1	0.928571428571428	protein coding gene	5	-	115297110	115300999	ENSMUSG00000009013	56455
Ercc3	excision repair cross-complementing rodent repair deficiency, comple	0.928571428571428	protein coding gene	18	+	32240300	32270151	ENSMUSG00000024382	13872
Gpr149	G protein-coupled receptor 149	0.928571428571428	protein coding gene	3	-	62529077	62605140	ENSMUSG00000043441	229357
Tram1l1	translocation associated membrane protein 1-like 1	0.928571428571428	protein coding gene	3	+	124320855	124324743	ENSMUSG00000044528	229801
Kprp	keratinocyte expressed, proline-rich	0.922172221673209	protein coding gene	3	-	92823074	92827247	ENSMUSG00000059832	433619
Mrgprb1	MAS-related GPR, member B1	0.913223320977314	protein coding gene	7	-	48444113	48456342	ENSMUSG00000070547	233231
Ptprr	protein tyrosine phosphatase, receptor type, R	0.913223320977314	protein coding gene	10	+	116018213	116274932	ENSMUSG00000020151	19279
Bbs12	Bardet-Biedl syndrome 12 (human)	0.904761904761905	protein coding gene	3	+	37312554	37321453	ENSMUSG00000051444	241950
Hdac1	histone deacetylase 1	0.904761904761905	protein coding gene	4	-	129516104	129542713	ENSMUSG00000028800	433759
Iqcc	IQ motif containing C	0.904761904761905	protein coding gene	4	-	129615126	129619099	ENSMUSG00000040795	230767
Mis18bp1	MIS18 binding protein 1	0.904761904761905	protein coding gene	12	-	65132734	65172604	ENSMUSG00000047534	217653
Moap1	modulator of apoptosis 1	0.904761904761905	protein coding gene	12	-	102742230	102743661	ENSMUSG00000096458	64113
Papola	poly (A) polymerase alpha	0.904761904761905	protein coding gene	12	+	105784694	105838944	ENSMUSG00000021111	18789
Sfxn4	sideroflexin 4	0.904761904761905	protein coding gene	19	-	60837277	60861479	ENSMUSG00000063698	94281
Smu1	smu-1 suppressor of mec-8 and unc-52 homolog (C. elegans)	0.904761904761905	protein coding gene	4	-	40736542	40757923	ENSMUSG00000028409	74255
Snx17	sorting nexin 17	0.904761904761905	protein coding gene	5	+	31193227	31199143	ENSMUSG00000029146	266781
Txlna	taxilin alpha	0.904761904761905	protein coding gene	4	-	129626083	129641065	ENSMUSG00000053841	109658
Vcpkmt	valosin containing protein lysine (K) methyltransferase	0.904761904761905	protein coding gene	12	-	69577628	69583028	ENSMUSG00000049882	207965
Vrk1	vaccinia related kinase 1	0.904761904761905	protein coding gene	12	+	106010228	106077426	ENSMUSG00000021115	22367
Nespas	neuroendocrine secretory protein antisense	0.902707567477393	antisense lncRNA gene	2	-	174281237	174295436	ENSMUSG00000086537	56802
Ccdc87	coiled-coil domain containing 87	0.898219696434944	protein coding gene	19	+	4839366	4842528	ENSMUSG00000067872	399599
Hist1h4c	histone cluster 1, H4c	0.886243433815811	protein coding gene	13	-	23698060	23698454	ENSMUSG00000060678	319155
0610038B21Rik	RIKEN cDNA 0610038B21 gene	0.880952380952381	antisense lncRNA gene	8	+	77517056	77518578	ENSMUSG00000097882	70345
Bloc1s2	biogenesis of lysosomal organelles complex-1, subunit 2	0.880952380952381	protein coding gene	19	-	44139266	44146446	ENSMUSG00000057506	73689
Cacna2d1	calcium channel, voltage-dependent, alpha2/delta subunit 1	0.880952380952381	protein coding gene	5	+	15934788	16374504	ENSMUSG00000040118	12293
Cd1d2	CD1d2 antigen	0.880952380952381	polymorphic pseudogene	3	+	86986551	86989780	ENSMUSG00000041750	12480
Cdk5r1	cyclin-dependent kinase 5, regulatory subunit 1 (p35)	0.880952380952381	protein coding gene	11	+	80477023	80481184	ENSMUSG00000048895	12569
Cetn2	centrin 2	0.880952380952381	protein coding gene	X	-	72913532	72918411	ENSMUSG00000031347	26370
Cpsf2	cleavage and polyadenylation specific factor 2	0.880952380952381	protein coding gene	12	+	101975764	102005993	ENSMUSG00000041781	51786
D430020J02Rik	RIKEN cDNA D430020J02 gene	0.880952380952381	ctional promoter lncRNA	12	-	116401947	116405161	ENSMUSG00000112980	319545
Grik2	glutamate receptor, ionotropic, kainate 2 (beta 2)	0.880952380952381	protein coding gene	10	-	49099460	49788766	ENSMUSG00000056073	14806
Mettl6	methyltransferase like 6	0.880952380952381	protein coding gene	14	-	31473578	31495040	ENSMUSG00000021891	67011
Neu3	neuraminidase 3	0.880952380952381	protein coding gene	7	-	99811439	99828417	ENSMUSG00000035239	50877
Pcbd2	pterin 4 alpha carbinolamine dehydratase/dimerization cofactor of he	0.880952380952381	protein coding gene	13	+	55727368	55776830	ENSMUSG00000021496	72562
Phtf1	putative homeodomain transcription factor 1	0.880952380952381	protein coding gene	3	+	103968110	104024598	ENSMUSG00000058388	18685
Serpina3h	serine (or cysteine) peptidase inhibitor, clade A, member 3H	0.880952380952381	pseudogene	12	+	104247905	104253669	ENSMUSG00000041449	546546
St8sia2	ST8 alpha-N-acetyl-neuraminide alpha-2,8-sialyltransferase 2	0.880952380952381	protein coding gene	7	-	73939119	74013690	ENSMUSG00000025789	20450
Tomm5	translocase of outer mitochondrial membrane 5	0.880952380952381	protein coding gene	4	-	45105208	45108114	ENSMUSG00000078713	68512
Wdr8		0.880952380952381							
Zmpste24	zinc metallopeptidase, STE24	0.880952380952381	protein coding gene	4	-	121059237	121098241	ENSMUSG00000043207	230709
Nutm1	NUT midline carcinoma, family member 1	0.87831006565368	protein coding gene	2	-	112247948	112259291	ENSMUSG00000041358	213765
Elavl2	ELAV like RNA binding protein 1	0.87287156094397	protein coding gene	4	-	91250763	91400785	ENSMUSG00000008489	15569
Soga3	SOGA family member 3	0.87287156094397	protein coding gene	10	+	29143839	29199630	ENSMUSG00000038916	67412
Cyp21a1	cytochrome P450, family 21, subfamily a, polypeptide 1	-0.87831006565368	protein coding gene	17	-	34801348	34804561	ENSMUSG00000024365	13079
Dlx1as	distal-less homeobox 1, antisense	-0.87831006565368	antisense lncRNA gene	2	-	71516454	71537891	ENSMUSG00000084946	111970
Lrrc66	leucine rich repeat containing 66	-0.87831006565368	protein coding gene	5	-	73606642	73632526	ENSMUSG00000067206	231296
Aak1	AP2 associated kinase 1	-0.880952380952381	protein coding gene	6	+	86849517	87003223	ENSMUSG00000057230	269774
Actg2	actin, gamma 2, smooth muscle, enteric	-0.880952380952381	protein coding gene	6	-	83512905	83536265	ENSMUSG00000059430	11468
Brwd3	bromodomain and WD repeat domain containing 3	-0.880952380952381	protein coding gene	X	-	108737016	108834372	ENSMUSG00000063663	382236
Cacnb1	calcium channel, voltage-dependent, beta 1 subunit	-0.880952380952381	protein coding gene	11	-	98001508	98023034	ENSMUSG00000020882	12295
Casq1	calsequestrin 1	-0.880952380952381	protein coding gene	1	-	172209894	172219868	ENSMUSG00000007122	12372
Cdk18	cyclin-dependent kinase 18	-0.880952380952381	protein coding gene	1	-	132112237	132139684	ENSMUSG00000026437	18557
Ceacam19	carcinoembryonic antigen-related cell adhesion molecule 19	-0.880952380952381	protein coding gene	7	-	19875742	19887965	ENSMUSG00000049848	319930
Dusp11	dual specificity phosphatase 11 (RNA/RNP complex 1-interacting)	-0.880952380952381	protein coding gene	6	-	85942268	85961667	ENSMUSG00000030002	72102
Fbxl3	F-box and leucine-rich repeat protein 3	-0.880952380952381	protein coding gene	14	-	103080239	103099566	ENSMUSG00000022124	50789
Ip6k2	inositol hexaphosphate kinase 2	-0.880952380952381	protein coding gene	9	+	108783796	108806337	ENSMUSG00000032599	76500
Klf1	Kruppel-like factor 1 (erythroid)	-0.880952380952381	protein coding gene	8	+	84901928	84905291	ENSMUSG00000054191	16596
Kmt2e	lysine (K)-specific methyltransferase 2E	-0.880952380952381	protein coding gene	5	+	23434441	23504235	ENSMUSG00000029004	69188
Ltbp3	latent transforming growth factor beta binding protein 3	-0.880952380952381	protein coding gene	19	+	5740904	5758532	ENSMUSG00000024940	16998
Mrpl14	mitochondrial ribosomal protein L14	-0.880952380952381	protein coding gene	17	+	45686322	45698495	ENSMUSG00000023939	68463
Ndfip2	Nedd4 family interacting protein 2	-0.880952380952381	protein coding gene	14	+	105258573	105309298	ENSMUSG00000053253	76273
Ndufa4l2	NADH dehydrogenase (ubiquinone) 1 alpha subcomplex, 4-like 2	-0.880952380952381	protein coding gene	10	+	127514967	127517154	ENSMUSG00000040280	407790
Ntrk3	neurotrophic tyrosine kinase, receptor, type 3	-0.880952380952381	protein coding gene	7	-	78175959	78738012	ENSMUSG00000059146	18213
Ptpn11	protein tyrosine phosphatase, non-receptor type 11	-0.880952380952381	protein coding gene	5	-	121130533	121191397	ENSMUSG00000043733	19247
Rbm20	RNA binding motif protein 20	-0.880952380952381	protein coding gene	19	+	53677306	53867080	ENSMUSG00000043639	73713
Slc25a43	solute carrier family 25, member 43	-0.880952380952381	protein coding gene	X	+	36743659	36777202	ENSMUSG00000037636	194744
Zfp467	zinc finger protein 467	-0.880952380952381	protein coding gene	6	-	48427697	48445825	ENSMUSG00000068551	68910
Epha8	Eph receptor A8	-0.887856006505722	protein coding gene	4	-	136929419	136956816	ENSMUSG00000028661	13842
Fam109a	family with sequence similarity 109, member A	-0.904761904761905	protein coding gene	5	+	121848984	121854632	ENSMUSG00000044134	231717
Itih2	inter-alpha trypsin inhibitor, heavy chain 2	-0.904761904761905	protein coding gene	2	-	10094593	10131396	ENSMUSG00000037254	16425
Kcnj8	potassium inwardly-rectifying channel, subfamily J, member 8	-0.904761904761905	protein coding gene	6	-	142564837	142571614	ENSMUSG00000030247	16523
Lamb2	laminin, beta 2	-0.904761904761905	protein coding gene	9	+	108479736	108490530	ENSMUSG00000052911	16779
Mxd1	MAX dimerization protein 1	-0.904761904761905	protein coding gene	6	-	86647042	86669161	ENSMUSG00000001156	17119
Plce1	phospholipase C, epsilon 1	-0.904761904761905	protein coding gene	19	+	38481109	38785030	ENSMUSG00000024998	74055
Sbds	SBDS ribosome maturation factor	-0.904761904761905	protein coding gene	5	-	130245731	130255530	ENSMUSG00000025337	66711
Scrn3	secernin 3	-0.904761904761905	protein coding gene	2	+	73312601	73337818	ENSMUSG00000008226	74616
Stfa2l1	stefin A2 like 1	-0.904761904761905	protein coding gene	16	+	36156811	36161948	ENSMUSG00000059657	268885
Vamp2	vesicle-associated membrane protein 2	-0.904761904761905	protein coding gene	11	+	69088490	69092384	ENSMUSG00000020894	22318
Zfand5	zinc finger, AN1-type domain 5	-0.904761904761905	protein coding gene	19	+	21272278	21282289	ENSMUSG00000024750	22682
Gm20139	predicted gene, 20139	-0.912870929175277	lincRNA gene	10	+	19141751	19206702	ENSMUSG00000111052	100504265
Gfi1b	growth factor independent 1B	-0.913223320977314	protein coding gene	2	-	28609455	28621982	ENSMUSG00000026815	14582
Arhgef10l	Rho guanine nucleotide exchange factor (GEF) 10-like	-0.928571428571428	protein coding gene	4	-	140514485	140666012	ENSMUSG00000040964	72754
Chd2	chromodomain helicase DNA binding protein 2	-0.928571428571428	protein coding gene	7	-	73426638	73541830	ENSMUSG00000078671	244059
Dusp13	dual specificity phosphatase 13	-0.928571428571428	protein coding gene	14	-	21733394	21751181	ENSMUSG00000021768	27389
Entpd3	ectonucleoside triphosphate diphosphohydrolase 3	-0.928571428571428	protein coding gene	9	+	120539818	120568318	ENSMUSG00000041608	215446
H2-Q1	histocompatibility 2, Q region locus 1	-0.928571428571428	protein coding gene	17	+	35320405	35325099	ENSMUSG00000079507	15006
Iqsec1	IQ motif and Sec7 domain 1	-0.928571428571428	protein coding gene	6	-	90656088	90988685	ENSMUSG00000034312	232227
Kmt2d	lysine (K)-specific methyltransferase 2D	-0.928571428571428	protein coding gene	15	-	98831672	98871204	ENSMUSG00000048154	381022
Myo1d	myosin ID	-0.928571428571428	protein coding gene	11	-	80482126	80780025	ENSMUSG00000035441	338367
Polr1d	polymerase (RNA) I polypeptide D	-0.928571428571428	protein coding gene	5	+	147077050	147111597	ENSMUSG00000029642	20018
Sos1	SOS Ras/Rac guanine nucleotide exchange factor 1	-0.928571428571428	protein coding gene	17	-	80393752	80480453	ENSMUSG00000024241	20662
Zscan25	zinc finger and SCAN domain containing 25	-0.928571428571428	protein coding gene	5	+	145280500	145291278	ENSMUSG00000070420	666311
Cux1	cut-like homeobox 1	-0.952380952380952	protein coding gene	5	-	136248135	136567490	ENSMUSG00000029705	13047
Duoxa1	dual oxidase maturation factor 1	-0.952380952380952	protein coding gene	2	-	122302191	122313730	ENSMUSG00000027224	213696
Gfpt1	glutamine fructose-6-phosphate transaminase 1	-0.952380952380952	protein coding gene	6	+	87042846	87092197	ENSMUSG00000029992	14583
Golga4	golgi autoantigen, golgin subfamily a, 4	-0.952380952380952	protein coding gene	9	+	118506267	118582519	ENSMUSG00000038708	54214
Setd2	SET domain containing 2	-0.952380952380952	protein coding gene	9	+	110532597	110618633	ENSMUSG00000044791	235626
Nat6	N-acetyltransferase 6	-0.976190476190476	protein coding gene	9	+	107578887	107584050	ENSMUSG00000079334	56441
Tnfrsf25	tumor necrosis factor receptor superfamily, member 25	-0.976190476190476	protein coding gene	4	+	152115934	152120119	ENSMUSG00000024793	85030
Zxdc	ZXD family zinc finger C	-0.976190476190476	protein coding gene	6	+	90369492	90403490	ENSMUSG00000034430	80292

Supplementary Table 2. List of enriched GO terms

GO ID	Pathway	FDR	Genes	Count
**GO.0006383**	transcription from RNA polymerase III promoter	1.83E-19	Crcp, Polr1c, Polr1d, Polr2e, Polr2f, Polr2h, Polr3b, Polr3c, Polr3d, Polr3e, Polr3f, Polr3h, Polr3k, Tbp	14
**GO.0006360**	transcription from RNA polymerase I promoter	1.98E-14	Gtf2h5, Polr1a, Polr1b, Polr1c, Polr1d, Polr1e, Polr2e, Polr2f, Polr2h, Twistnb, Znrd1	11
**GO.0016070**	RNA metabolic process	4.05E-13	Chd2, Cpsf1, Cpsf3, Cpsf4, Crcp, Cstf1, Cstf2, Ctnnb1, Cux1, Dmrt2, Dusp11, Dynll1, Ercc2, Ercc3, Fip1l1, Gfi1b, Gtf2a2, Gtf2b, Gtf2e1, Gtf2e2, Gtf2h3, Gtf2h4, Gtf2h5, Hdac1, Klf1, Mll2, Mll5, Mnat1, Mxd1, Papola, Phtf1, Polr1a, Polr1b, Polr1c, Polr1d, Polr1e, Polr2e, Polr2f, Polr2h, Polr3b, Polr3c, Polr3d, Polr3e, Polr3f, Polr3h, Polr3k, Ppih, Prpf4, Rbm20, Sbds, Setd2, Sin3a, Taf1, Taf7, Twistnb, Wdr33, Zfp467, Znrd1, Zxdc	59
**GO.0010467**	gene expression	3.11E-12	Chd2, Cpsf1, Cpsf3, Cpsf4, Crcp, Cstf1, Cstf2, Ctnnb1, Cux1, Dmrt2, Dynll1, Ercc2, Ercc3, Fip1l1, Gfi1b, Gtf2a2, Gtf2b, Gtf2e1, Gtf2e2, Gtf2h3, Gtf2h4, Gtf2h5, Hdac1, Klf1, Mll2, Mll5, Mnat1, Mrpl14, Mrpl15, Mxd1, Papola, Phtf1, Polr1a, Polr1b, Polr1c, Polr1d, Polr1e, Polr2e, Polr2f, Polr2h, Polr3b, Polr3c, Polr3d, Polr3e, Polr3f, Polr3h, Polr3k, Ppih, Prpf4, Rbm20, Sbds, Setd2, Sin3a, Taf1, Taf7, Twistnb, Wdr33, Zfp467, Zmpste24, Znrd1, Zxdc	61
**GO.0006139**	nucleobase-containing compound metabolic process	7.52E-12	Cetn2, Chd2, Cps1, Cpsf1, Cpsf3, Cpsf4, Crcp, Cstf1, Cstf2, Ctnnb1, Cux1, Dmrt2, Dusp11, Dynll1, Ercc2, Ercc3, Fip1l1, Gfi1b, Gfpt1, Gtf2a2, Gtf2b, Gtf2e1, Gtf2e2, Gtf2h3, Gtf2h5, Hdac1, Klf1, Mll2, Mll5, Mnat1, Mxd1, Ndufv2, Papola, Phtf1, Polr1a, Polr1b, Polr1c, Polr1d, Polr1e, Polr2e, Polr2f, Polr2h, Polr3b, Polr3c, Polr3d, Polr3e, Polr3f, Polr3h, Polr3k, Ppih, Prpf4, Rbm20, Sbds, Setd2, Sin3a, Taf1, Taf7, Twistnb, Wdr33, Xpc, Zfp467,	65
**GO.0034641**	cellular nitrogen compound metabolic process	7.6E-12	Cetn2, Chd2, Cps1, Cpsf1, Cpsf3, Cpsf4, Crcp, Cstf1, Cstf2, Ctnnb1, Cux1, Dmrt2, Dusp11, Dynll1, Ercc2, Ercc3, Fip1l1, Gfi1b, Gfpt1, Gtf2a2, Gtf2b, Gtf2e1, Gtf2e2, Gtf2h3, Gtf2h5, Hdac1, Klf1, Mll2, Mll5, Mnat1, Mxd1, Ndufv2, Papola, Phtf1, Polr1a, Polr1b, Polr1c, Polr1d, Polr1e, Polr2e, Polr2f, Polr2h, Polr3b, Polr3c, Polr3d, Polr3e, Polr3f, Polr3h, Polr3k, Ppih, Prpf4, Rbm20, Sbds, Setd2, Sin3a, Taf1, Taf7, Twistnb, Wdr33, Xpc, Zfp467,	70
**GO.0006725**	cellular aromatic compound metabolic process	8.94E-12	Cetn2, Chd2, Cps1, Cpsf1, Cpsf3, Cpsf4, Crcp, Cstf1, Cstf2, Ctnnb1, Cux1, Dmrt2, Dusp11, Dynll1, Ercc2, Ercc3, Fip1l1, Gfi1b, Gfpt1, Gtf2a2, Gtf2b, Gtf2e1, Gtf2e2, Gtf2h3, Gtf2h5, Hdac1, Klf1, Mll2, Mll5, Mnat1, Mxd1, Ndufv2, Papola, Pcbd2, Phtf1, Polr1a, Polr1b, Polr1c, Polr1d, Polr1e, Polr2e, Polr2f, Polr2h, Polr3b, Polr3c, Polr3d, Polr3e, Polr3f, Polr3h, Polr3k, Ppih, Prpf4, Rbm20, Sbds, Setd2, Sin3a, Taf1, Taf7, Twistnb,	66
**GO.0046483**	heterocycle metabolic process	8.94E-12	Cetn2, Chd2, Cps1, Cpsf1, Cpsf3, Cpsf4, Crcp, Cstf1, Cstf2, Ctnnb1, Cux1, Dmrt2, Dusp11, Dynll1, Ercc2, Ercc3, Fip1l1, Gfi1b, Gfpt1, Gtf2a2, Gtf2b, Gtf2e1, Gtf2e2, Gtf2h3, Gtf2h5, Hdac1, Klf1, Mll2, Mll5, Mnat1, Mxd1, Ndufv2, Papola, Pcbd2, Phtf1, Polr1a, Polr1b, Polr1c, Polr1d, Polr1e, Polr2e, Polr2f, Polr2h, Polr3b, Polr3c, Polr3d, Polr3e, Polr3f, Polr3h, Polr3k, Ppih, Prpf4, Rbm20, Sbds, Setd2, Sin3a, Taf1, Taf7, Twistnb,	66
**GO.1901360**	organic cyclic compound metabolic process	8.94E-12	Baat, Cetn2, Chd2, Cps1, Cpsf1, Cpsf3, Cpsf4, Crcp, Cstf1, Cstf2, Ctnnb1, Cux1, Cyp21a1, Dmrt2, Dusp11, Dynll1, Ercc2, Ercc3, Fip1l1, Gfi1b, Gfpt1, Gtf2a2, Gtf2b, Gtf2e1, Gtf2e2, Gtf2h3, Gtf2h5, Hdac1, Klf1, Mll2, Mll5, Mnat1, Mxd1, Ndufv2, Papola, Pcbd2, Phtf1, Polr1a, Polr1b, Polr1c, Polr1d, Polr1e, Polr2e, Polr2f, Polr2h, Polr3b, Polr3c, Polr3d, Polr3e, Polr3f, Polr3h, Polr3k, Ppih, Prpf4, Rbm20, Sbds, Setd2, Sin3a, Taf1, Taf7, Twistnb, Wdr33, Xpc, Zfp467, Znrd1, Zxdc, mt-Nd4, mt-Nd5	68
**GO.0006807**	nitrogen compound metabolic process	9.01E-12	Baat, Cetn2, Chd2, Cps1, Cpsf1, Cpsf3, Cpsf4, Crcp, Cstf1, Cstf2, Ctnnb1, Cux1, Dmrt2, Dusp11, Dynll1, Ercc2, Ercc3, Fip1l1, Gfi1b, Gfpt1, Gtf2a2, Gtf2b, Gtf2e1, Gtf2e2, Gtf2h3, Gtf2h5, Hdac1, Itih2, Klf1, Mll2, Mll5, Mnat1, Mrpl14, Mrpl15, Mxd1, Ndufv2, Neu3, Papola, Pcbd2, Phtf1, Polr1a, Polr1b, Polr1c, Polr1d, Polr1e, Polr2e, Polr2f, Polr2h, Polr3b, Polr3c, Polr3d, Polr3e, Polr3f, Polr3h, Polr3k, Ppih, Prpf4, Rbm20, Sbds, Setd2, Sin3a, St8sia2, Taf1, Taf7, Twistnb, Wdr33, Xpc, Zfp467, Znrd1, Zxdc, mt-Nd4, mt-	72
**GO.0090304**	nucleic acid metabolic process	1.28E-11	Cetn2, Chd2, Cpsf1, Cpsf3, Cpsf4, Crcp, Cstf1, Cstf2, Ctnnb1, Cux1, Dmrt2, Dusp11, Dynll1, Ercc2, Ercc3, Fip1l1, Gfi1b, Gtf2a2, Gtf2b, Gtf2e1, Gtf2e2, Gtf2h3, Gtf2h5, Hdac1, Klf1, Mll2, Mll5, Mnat1, Mxd1, Papola, Phtf1, Polr1a, Polr1b, Polr1c, Polr1d, Polr1e, Polr2e, Polr2f, Polr2h, Polr3b, Polr3c, Polr3d, Polr3e, Polr3f, Polr3h, Polr3k, Ppih, Prpf4, Rbm20, Sbds, Setd2, Sin3a, Taf1, Taf7, Twistnb, Wdr33, Xpc, Zfp467, Znrd1, Zxdc	60
**GO.0006351**	transcription, DNA-templated	1.14E-10	Chd2, Crcp, Ctnnb1, Cux1, Dmrt2, Dynll1, Ercc2, Ercc3, Gfi1b, Gtf2a2, Gtf2b, Gtf2e1, Gtf2e2, Gtf2h3, Gtf2h4, Gtf2h5, Hdac1, Klf1, Mll2, Mll5, Mnat1, Mxd1, Phtf1, Polr1a, Polr1b, Polr1c, Polr1d, Polr1e, Polr2e, Polr2f, Polr2h, Polr3b, Polr3c, Polr3d, Polr3e, Polr3f, Polr3h, Polr3k, Setd2, Sin3a, Taf1, Taf7, Twistnb, Zfp467, Znrd1, Zxdc	46
**GO.1901362**	organic cyclic compound biosynthetic process	1.16E-10	Baat, Chd2, Cps1, Crcp, Ctnnb1, Cux1, Cyp21a1, Dmrt2, Dynll1, Ercc2, Ercc3, Gfi1b, Gfpt1, Gtf2a2, Gtf2b, Gtf2e1, Gtf2e2, Gtf2h3, Gtf2h4, Gtf2h5, Hdac1, Klf1, Mll2, Mll5, Mnat1, Mxd1, Pcbd2, Phtf1, Polr1a, Polr1b, Polr1c, Polr1d, Polr1e, Polr2e, Polr2f, Polr2h, Polr3b, Polr3c, Polr3d, Polr3e, Polr3f, Polr3h, Polr3k, Setd2, Sin3a, Taf1, Taf7, Twistnb, Zfp467, Znrd1,	51
**GO.0018130**	heterocycle biosynthetic process	2.79E-10	Chd2, Cps1, Crcp, Ctnnb1, Cux1, Dmrt2, Dynll1, Ercc2, Ercc3, Gfi1b, Gfpt1, Gtf2a2, Gtf2b, Gtf2e1, Gtf2e2, Gtf2h3, Gtf2h4, Gtf2h5, Hdac1, Klf1, Mll2, Mll5, Mnat1, Mxd1, Pcbd2, Phtf1, Polr1a, Polr1b, Polr1c, Polr1d, Polr1e, Polr2e, Polr2f, Polr2h, Polr3b, Polr3c, Polr3d, Polr3e, Polr3f, Polr3h, Polr3k, Setd2, Sin3a, Taf1, Taf7, Twistnb, Zfp467, Znrd1, Zxdc	49
**GO.0006352**	DNA-templated transcription, initiation	2.97E-10	Crcp, Gtf2a1, Gtf2a2, Gtf2b, Gtf2e1, Gtf2e2, Polr1e, Polr3h, Taf1, Taf7, Tbp	11
**GO.0019438**	aromatic compound biosynthetic process	2.97E-10	Cad, Chd2, Crcp, Ctnnb1, Cux1, Dmrt2, Dynll1, Ercc2, Ercc3, Gfi1b, Gfpt1, Gtf2a2, Gtf2b, Gtf2e1, Gtf2e2, Gtf2h3, Gtf2h4, Gtf2h5, Hdac1, Klf1, Mll2, Mll5, Mnat1, Mxd1, Pcbd2, Phtf1, Polr1a, Polr1b, Polr1c, Polr1d, Polr1e, Polr2e, Polr2f, Polr2h, Polr3b, Polr3c, Polr3d, Polr3e, Polr3f, Polr3h, Polr3k, Setd2, Sin3a, Taf1, Taf7, Twistnb, Zfp467, Znrd1, Zxdc	49

GO ID	Pathway	FDR	Genes	Count
**GO.0034654**	nucleobase-containing compound biosynthetic proces	3.28E-10	Cad, Chd2, Crcp, Ctnnb1, Cux1, Dmrt2, Dynll1, Ercc2, Ercc3, Gfi1b, Gfpt1, Gtf2a2, Gtf2b, Gtf2e1, Gtf2e2, Gtf2h3, Gtf2h4, Gtf2h5, Hdac1, Klf1, Mll2, Mll5, Mnat1, Mxd1, Phtf1, Polr1a, Polr1b, Polr1c, Polr1d, Polr1e, Polr2e, Polr2f, Polr2h, Polr3b, Polr3c, Polr3d, Polr3e, Polr3f, Polr3h, Polr3k, Setd2, Sin3a, Taf1, Taf7, Twistnb, Zfp467, Znrd1, Zxdc	48
**GO.0044271**	cellular nitrogen compound biosynthetic process	8.79E-10	Chd2, Cps1, Crcp, Ctnnb1, Cux1, Dmrt2, Dynll1, Ercc2, Ercc3, Gfi1b, Gfpt1, Gtf2a2, Gtf2b, Gtf2e1, Gtf2e2, Gtf2h3, Gtf2h4, Gtf2h5, Hdac1, Klf1, Mll2, Mll5, Mnat1, Mrpl14, Mrpl15, Mxd1, Pcbd2, Phtf1, Polr1a, Polr1b, Polr1c, Polr1d, Polr1e, Polr2e, Polr2f, Polr2h, Polr3b, Polr3c, Polr3d, Polr3e, Polr3f, Polr3h, Polr3k, Setd2, Sin3a, St8sia2, Taf1, Taf7, Twistnb, Zfp467,	52
**GO.0000183**	chromatin silencing at rDNA	8.92E-09	Hdac1, Polr1a, Polr1b, Polr1c, Polr1d, Polr1e, Sin3a, Znrd1	8
**GO.0006342**	chromatin silencing	1.09E-08	Hdac1, Mll2, Polr1a, Polr1b, Polr1c, Polr1d, Polr1e, Sin3a, Znrd1	9
**GO.0044260**	cellular macromolecule metabolic process	2.52E-08	Aak1, B2m, Cad, Cdk18, Cetn2, Chd2, Cps1, Cpsf1, Cpsf3, Cpsf4, Crcp, Cstf1, Cstf2, Ctnnb1, Cux1, Dmrt2, Dusp13, Dynll1, Epha8, Fam109a, Fbxl3, Fip1l1, Gab1, Gfi1b, Grb2, Gtf2a2, Gtf2b, Gtf2e1, Gtf2e2, Gtf2h5, Hdac1, Klf1, Mettl21d, Mll2, Mll5, Mrpl14, Mrpl15, Mxd1, Ndufab1, Ntrk3, Papola, Phtf1, Polr1a, Polr1b, Polr1c, Polr1d, Polr1e, Polr2e, Polr2f, Polr2h, Polr3b, Polr3c, Polr3d, Polr3e, Polr3f, Polr3h, Polr3k, Ppih, Ppp4c, Prpf4, Ptpn11, Ptprr, Rbm20, Sbds, Setd2, Sin3a, St8sia2, Taf7, Twistnb, Vrk1, Wdr33,	75
**GO.0008152**	metabolic process	1.26E-07	Aak1, Arl1, B2m, Baat, Cdk18, Cetn2, Chd2, Cpsf1, Cpsf3, Cpsf4, Crcp, Crhbp, Cstf1, Cstf2, Ctnnb1, Cux1, Cyp21a1, Dmrt2, Duoxa1, Dusp13, Dynll1, Epha8, Fam109a, Fbxl3, Fip1l1, Gab1, Gfi1b, Gfpt1, Grb2, Gtf2a2, Gtf2b, Gtf2e1, Gtf2e2, Gtf2h5, Hdac1, Itih2, Klf1, Mettl21d, Mettl6, Mll2, Mll5, Mrpl14, Mrpl15, Mxd1, Nat6, Ndufa12, Ndufa13, Ndufa9, Ndufab1, Ndufb2, Ndufb5, Ndufb6, Ndufv2, Neu3, Ntrk3, Papola, Pcbd2, Phtf1, Plce1, Polr1a, Polr1b, Polr1c, Polr1d, Polr1e, Polr2e, Polr2f, Polr2h, Polr3b, Polr3c, Polr3d, Polr3e, Polr3f, Polr3h, Polr3k, Ppih, Ppp4c, Prpf4, Ptpn11, Ptprr, Rbm20, Sbds, Scrn3, Setd2, Sin3a, St8sia2, Taf7, Twistnb, Vrk1	94
**GO.0034645**	cellular macromolecule biosynthetic process	1.44E-07	Chd2, Crcp, Ctnnb1, Cux1, Dmrt2, Dynll1, Ercc2, Ercc3, Gfi1b, Gtf2a2, Gtf2b, Gtf2e1, Gtf2e2, Gtf2h3, Gtf2h4, Gtf2h5, Hdac1, Klf1, Mll2, Mll5, Mnat1, Mrpl14, Mrpl15, Mxd1, Phtf1, Polr1a, Polr1b, Polr1c, Polr1d, Polr1e, Polr2e, Polr2f, Polr2h, Polr3b, Polr3c, Polr3d, Polr3e, Polr3f, Polr3h, Polr3k, Setd2, Sin3a, St8sia2, Taf1, Taf7, Twistnb, Zfp467, Znrd1,	49
**GO.0044237**	cellular metabolic process	2.4E-07	Aak1, Arl1, B2m, Baat, Cdk18, Cetn2, Chd2, Cps1, Cpsf1, Cpsf3, Cpsf4, Crcp, Cstf1, Cstf2, Ctnnb1, Cux1, Dmrt2, Duoxa1, Dusp13, Dynll1, Epha8, Fam109a, Fbxl3, Fip1l1, Gab1, Gfi1b, Gfpt1, Grb2, Gtf2a2, Gtf2b, Gtf2e1, Gtf2e2, Gtf2h5, Hdac1, Klf1, Mettl21d, Mll2, Mll5, Mrpl14, Mrpl15, Mxd1, Ndufa9, Ndufab1, Ndufv2, Neu3, Ntrk3, Papola, Pcbd2, Phtf1, Polr1a, Polr1b, Polr1c, Polr1d, Polr1e, Polr2e, Polr2f, Polr2h, Polr3b, Polr3c, Polr3d, Polr3e, Polr3f, Polr3h, Polr3k, Ppih, Ppp4c, Prpf4, Ptpn11, Ptprr, Rbm20, Sbds, Setd2, Sin3a, St8sia2, Taf7, Twistnb, Vrk1, Wdr33, Xpc	84
**GO.1901576**	organic substance biosynthetic process	2.45E-07	Baat, Chd2, Cps1, Crcp, Ctnnb1, Cux1, Cyp21a1, Dmrt2, Dynll1, Ercc2, Ercc3, Gfi1b, Gfpt1, Gtf2a2, Gtf2b, Gtf2e1, Gtf2e2, Gtf2h3, Gtf2h4, Gtf2h5, Hdac1, Klf1, Mll2, Mll5, Mnat1, Mrpl14, Mrpl15, Mxd1, Ndufa9, Ndufab1, Pcbd2, Phtf1, Polr1a, Polr1b, Polr1c, Polr1d, Polr1e, Polr2e, Polr2f, Polr2h, Polr3b, Polr3c, Polr3d, Polr3e, Polr3f, Polr3h, Polr3k, Setd2, Sin3a, St8sia2, Taf1, Taf7, Twistnb, Zfp467, Znrd1, Zxdc	56
**GO.0044249**	cellular biosynthetic process	3.38E-07	Baat, Chd2, Cps1, Crcp, Ctnnb1, Cux1, Dmrt2, Dynll1, Ercc2, Ercc3, Gfi1b, Gfpt1, Gtf2a2, Gtf2b, Gtf2e1, Gtf2e2, Gtf2h3, Gtf2h4, Gtf2h5, Hdac1, Klf1, Mll2, Mll5, Mnat1, Mrpl14, Mrpl15, Mxd1, Ndufa9, Ndufab1, Pcbd2, Phtf1, Polr1a, Polr1b, Polr1c, Polr1d, Polr1e, Polr2e, Polr2f, Polr2h, Polr3b, Polr3c, Polr3d, Polr3e, Polr3f, Polr3h, Polr3k, Setd2, Sin3a, St8sia2, Taf1,	55
**GO.0050789**	regulation of biological process	3.41E-07	Aak1, Actg2, Aipl1, Arhgef10l, B2m, Bbs12, Bloc1s2b, Brwd3, Cacna2d1, Cacnb1, Cd1d2, Cdh2, Cdk18, Cdk5, Cdk5r1, Cetn2, Chd2, Cps1, Crcp, Crhbp, Ctnnb1, Cux1, Dmrt2, Duoxa1, Dynll1, Epha8, Ercc2, Ercc3, Fam109a, Fbxl3, Gab1, Gfi1b, Gfpt1, Golga4, Gpr149, Grb2, Gtf2a1, Gtf2a2, Gtf2b, Gtf2e1, Gtf2e2, Gtf2h3, Gtf2h4, Gtf2h5, Hdac1, Ip6k2, Iqsec1, Itih2, Kcnj8, Klf1, Ltbp3, Mll2, Mll5, Mrgprb1, Mxd1, Myo1d, Ndfip2, Ndufa13, Papola, Pcbd2, Phtf1, Polr1a, Polr1b, Polr1c, Polr1d, Polr1e, Polr3b, Polr3c, Polr3d, Polr3f, Ppih, Ppp4c, Ptpn11, Ptprr, Rbm20, Sbds, Setd2, Sfxn4, Sin3a, Snap25, Snx17, Soga3, Stfa2l1, Stx1a, Syt1, Taf1, Tbp, Txlna, Vrk1	94
**GO.0043170**	macromolecule metabolic process	4.44E-07	Aak1, B2m, Cad, Cdk18, Cetn2, Chd2, Cpsf1, Cpsf3, Cpsf4, Crcp, Cstf1, Cstf2, Ctnnb1, Cux1, Dmrt2, Dusp13, Dynll1, Epha8, Fam109a, Fbxl3, Fip1l1, Gab1, Gfi1b, Grb2, Gtf2a2, Gtf2b, Gtf2e1, Gtf2e2, Gtf2h5, Hdac1, Itih2, Klf1, Mettl21d, Mll2, Mll5, Mrpl14, Mrpl15, Mxd1, Ndufab1, Ntrk3, Papola, Phtf1, Polr1a, Polr1b, Polr1c, Polr1d, Polr1e, Polr2e, Polr2f, Polr2h, Polr3b, Polr3c, Polr3d, Polr3e, Polr3f, Polr3h, Polr3k, Ppih, Ppp4c, Prpf4, Ptpn11, Ptprr, Rbm20, Sbds, Scrn3, Setd2, Sin3a, St8sia2, Taf7, Twistnb, Vrk1,	76
**GO.0071704**	organic substance metabolic process	5.65E-07	Aak1, B2m, Baat, Cdk18, Cetn2, Chd2, Cpsf1, Cpsf3, Cpsf4, Crcp, Cstf1, Cstf2, Ctnnb1, Cux1, Cyp21a1, Dmrt2, Dusp13, Dynll1, Epha8, Fam109a, Fbxl3, Fip1l1, Gab1, Gfi1b, Gfpt1, Grb2, Gtf2a2, Gtf2b, Gtf2e1, Gtf2e2, Gtf2h5, Hdac1, Ip6k2, Itih2, Klf1, Mettl21d, Mll2, Mll5, Mrpl14, Mrpl15, Mxd1, Ndufa9, Ndufab1, Ndufv2, Neu3, Ntrk3, Papola, Pcbd2, Phtf1, Plce1, Polr1a, Polr1b, Polr1c, Polr1d, Polr1e, Polr2e, Polr2f, Polr2h, Polr3b, Polr3c, Polr3d, Polr3e, Polr3f, Polr3h, Polr3k, Ppih, Ppp4c, Prpf4, Ptpn11, Ptprr, Rbm20, Sbds, Scrn3, Setd2, Sin3a, St8sia2, Taf7, Twistnb, Vrk1	86
**GO.0040029**	regulation of gene expression, epigenetic	9.62E-06	Ctnnb1, Hdac1, Mll2, Mll5, Polr1a, Polr1b, Polr1c, Polr1d, Polr1e, Sin3a, Znrd1	11
**GO.0044238**	primary metabolic process	1.23E-05	Aak1, B2m, Baat, Cdk18, Cetn2, Chd2, Cpsf1, Cpsf3, Cpsf4, Crcp, Cstf1, Cstf2, Ctnnb1, Cux1, Cyp21a1, Dmrt2, Dusp13, Dynll1, Epha8, Fbxl3, Fip1l1, Gab1, Gfi1b, Gfpt1, Gtf2a2, Gtf2b, Gtf2e1, Gtf2e2, Gtf2h5, Hdac1, Ip6k2, Klf1, Mettl21d, Mll2, Mll5, Mrpl14, Mrpl15, Mxd1, Ndufab1, Ndufv2, Neu3, Ntrk3, Papola, Phtf1, Plce1, Polr1a, Polr1b, Polr1c, Polr1d, Polr1e, Polr2e, Polr2f, Polr2h, Polr3b, Polr3c, Polr3d, Polr3e, Polr3f, Polr3h, Polr3k, Ppih, Ppp4c, Prpf4, Ptpn11, Ptprr, Rbm20, Sbds, Scrn3, Setd2, Sin3a, St8sia2, Taf7, Twistnb, Vrk1, Wdr33, Xpc, Zfp467, Znrd1, Zxdc, mt-	81
**GO.0006289**	nucleotide-excision repair	2.09E-05	Cetn2, Ercc2, Ercc3, Gtf2h3, Gtf2h4, Gtf2h5, Xpc	7

GO ID	Pathway	FDR	Genes	Count
**GO.0009987**	cellular process	2.89E-05	Aak1, B2m, Baat, Bbs12, Bloc1s2b, Brwd3, Cacna2d1, Cacnb1, Casq1, Ccdc124, Ccdc66, Ccdc87, Cdh2, Cdk18, Cdk5r1, Cetn2, Chd2, Cpsf1, Cpsf3, Cpsf4, Crcp, Crhbp, Cstf1, Cstf2, Ctnnb1, Cux1, Dmrt2, Duoxa1, Dusp13, Epha8, Fam109a, Fbxl3, Fip1l1, Gab1, Gfi1b, Gfpt1, Golga4, Gpr149, Gtf2a2, Gtf2b, Gtf2e1, Gtf2e2, Gtf2h5, Hdac1, Iqsec1, Kcnj8, Klf1, Lamb2, Ltbp3, Mettl21d, Mis18bp1, Mll2, Mll5, Mrgprb1, Mrpl14, Mrpl15, Mxd1, Ndfip2, Ndufa13, Ndufa9, Ndufab1, Ndufv2, Neu3, Papola, Pcbd2, Phtf1, Polr1a, Polr1b, Polr1c, Polr1d, Polr1e, Polr2e, Polr2f, Polr2h, Polr3b, Polr3c, Polr3d, Polr3e, Polr3f, Polr3h, Polr3k, Ppih, Prpf4, Ptpn11, Rbm20, Sbds, Scrn3, Setd2, Sfxn4, Sin3a, Slc25a43, Snap25, Snx17, St8sia2, Syt1, [illegible]	108
**GO.0065007**	biological regulation	4.38E-05	Aak1, Actg2, Aipl1, Arhgef10l, Bbs12, Bloc1s2b, Brwd3, Cacna2d1, Cacnb1, Cad, Cd1d2, Cdh2, Cdk18, Cdk5, Cdk5r1, Cetn2, Chd2, Crcp, Crhbp, Ctnnb1, Cux1, Dmrt2, Duoxa1, Dynll1, Epha8, Ercc2, Ercc3, Fam109a, Fbxl3, Gab1, Gfi1b, Gfpt1, Golga4, Gpr149, Grb2, Gtf2a1, Gtf2a2, Gtf2b, Gtf2e1, Gtf2e2, Gtf2h3, Gtf2h4, Gtf2h5, Hdac1, Ip6k2, Iqsec1, Itih2, Klf1, Ltbp3, Mll2, Mll5, Mrgprb1, Mxd1, Myo1d, Ndfip2, Ndufa13, Papola, Pcbd2, Phtf1, Polr1a, Polr1b, Polr1c, Polr1d, Polr1e, Polr3b, Polr3c, Polr3d, Polr3f, Ppih, Ppp4c, Ptpn11, Ptprr, Rbm20, Sbds, Setd2, Sfxn4, Sin3a, Snap25, Snx17, Soga3, Stfa2l1, Syt1, Taf1, Tbp, Txlna, Vrk1, Xpc, Zfand5, Zfp467, Znrd1, Zxdc	91
**GO.0051252**	regulation of RNA metabolic process	5.17E-05	Bloc1s2b, Brwd3, Cdk5, Chd2, Ctnnb1, Cux1, Dmrt2, Dynll1, Ercc2, Ercc3, Gfi1b, Gtf2a1, Gtf2a2, Gtf2b, Gtf2e1, Gtf2e2, Gtf2h3, Gtf2h4, Gtf2h5, Hdac1, Klf1, Mll2, Mll5, Mnat1, Mxd1, Ndufa13, Ntrk3, Papola, Pcbd2, Phtf1, Polr1a, Polr1b, Polr1c, Polr1d, Polr1e, Rbm20, Setd2, Sfxn4, Sin3a, Taf1,	44
**GO.0031323**	regulation of cellular metabolic process	6.9E-05	Aipl1, Bloc1s2b, Brwd3, Cd1d2, Cdh2, Cdk5, Chd2, Ctnnb1, Cux1, Dmrt2, Duoxa1, Dynll1, Epha8, Ercc2, Ercc3, Gab1, Gfi1b, Grb2, Grik2, Gtf2a1, Gtf2a2, Gtf2b, Gtf2e1, Gtf2e2, Gtf2h3, Gtf2h4, Gtf2h5, Hdac1, Itih2, Klf1, Mll2, Mll5, Mnat1, Mxd1, Myo1d, Ndfip2, Ndufa13, Papola, Pcbd2, Phtf1, Plce1, Polr1a, Polr1b, Polr1c, Polr1d, Polr1e, Ppp4c, Ptpn11, Ptprr, Rbm20, Setd2, Sfxn4, Sin3a, Soga3, Stfa2l1, Taf1, Tbp, Txlna, Zfp467, Znrd1, Zxdc	61
**GO.0051607**	defense response to virus	6.97E-05	Crcp, Kcnj8, Polr3b, Polr3c, Polr3d, Polr3e, Polr3f, Polr3h, Polr3k	9
**GO.0006366**	transcription from RNA polymerase II promoter	7.46E-05	Cux1, Dmrt2, Ercc2, Ercc3, Gtf2a2, Gtf2e1, Gtf2e2, Gtf2h3, Gtf2h4, Mnat1, Polr2e, Polr2f, Polr2h, Setd2, Taf7, Tbp	16
**GO.0019219**	regulation of nucleobase-containing compound metab	7.7E-05	Aipl1, Bloc1s2b, Brwd3, Cdk5, Chd2, Ctnnb1, Cux1, Dmrt2, Dynll1, Ercc2, Ercc3, Gfi1b, Gtf2a1, Gtf2a2, Gtf2b, Gtf2e1, Gtf2e2, Gtf2h3, Gtf2h4, Gtf2h5, Hdac1, Klf1, Mll2, Mll5, Mnat1, Mxd1, Ndufa13, Ntrk3, Papola, Pcbd2, Phtf1, Polr1a, Polr1b, Polr1c, Polr1d, Polr1e, Ppp4c, Rbm20, Setd2, Sfxn4, Sin3a, Taf1, Tbp, Zfp467, Znrd1, Zxdc	46
**GO.0019222**	regulation of metabolic process	9.85E-05	Actg2, Aipl1, Arhgef10l, Arl1, Bloc1s2b, Brwd3, Cd1d2, Cdh2, Cdk5, Chd2, Ctnnb1, Cux1, Dmrt2, Duoxa1, Dynll1, Epha8, Ercc2, Ercc3, Gab1, Gfi1b, Gfpt1, Grb2, Grik2, Gtf2a1, Gtf2a2, Gtf2b, Gtf2e1, Gtf2e2, Gtf2h3, Gtf2h4, Gtf2h5, Hdac1, Iqsec1, Itih2, Klf1, Mll2, Mll5, Mnat1, Mxd1, Myo1d, Ndfip2, Ndufa13, Papola, Pcbd2, Phtf1, Plce1, Polr1a, Polr1b, Polr1c, Polr1d, Polr1e, Ppp4c, Ptpn11, Ptprr, Rbm20, Setd2, Sfxn4, Sin3a, Soga3, Stfa2l1,	66
**GO.0060255**	regulation of macromolecule metabolic process	0.000107	Actg2, Bloc1s2b, Brwd3, Cd1d2, Cdh2, Cdk5, Chd2, Ctnnb1, Cux1, Dmrt2, Dynll1, Epha8, Ercc2, Ercc3, Gab1, Gfi1b, Gfpt1, Grb2, Grik2, Gtf2a1, Gtf2a2, Gtf2b, Gtf2e1, Gtf2e2, Gtf2h3, Gtf2h4, Gtf2h5, Hdac1, Itih2, Klf1, Mll2, Mll5, Mnat1, Mxd1, Myo1d, Ndfip2, Ndufa13, Papola, Pcbd2, Phtf1, Plce1, Polr1a, Polr1b, Polr1c, Polr1d, Polr1e, Ppp4c, Ptpn11, Ptprr, Rbm20, Setd2, Sfxn4, Sin3a, Stfa2l1, Taf1, Tbp, Zfp467, Znrd1, Zxdc	59
**GO.0080090**	regulation of primary metabolic process	0.000113	Aipl1, Bloc1s2b, Brwd3, Cd1d2, Cdh2, Cdk5, Chd2, Ctnnb1, Cux1, Dmrt2, Duoxa1, Dynll1, Epha8, Ercc2, Ercc3, Gab1, Gfi1b, Grb2, Grik2, Gtf2a1, Gtf2a2, Gtf2b, Gtf2e1, Gtf2e2, Gtf2h3, Gtf2h4, Gtf2h5, Hdac1, Itih2, Klf1, Mll2, Mll5, Mnat1, Mxd1, Myo1d, Ndfip2, Ndufa13, Papola, Pcbd2, Phtf1, Plce1, Polr1a, Polr1b, Polr1c, Polr1d, Polr1e, Ppp4c, Ptpn11, Ptprr, Rbm20, Setd2, Sfxn4, Sin3a, Stfa2l1, Taf1, Tbp, Zfp467, Znrd1, Zxdc	59
**GO.0006355**	regulation of transcription, DNA-templated	0.000119	Bloc1s2b, Brwd3, Cdk5, Chd2, Ctnnb1, Cux1, Dmrt2, Dynll1, Ercc2, Ercc3, Gfi1b, Gtf2a1, Gtf2a2, Gtf2b, Gtf2e1, Gtf2e2, Gtf2h3, Gtf2h4, Gtf2h5, Hdac1, Klf1, Mll2, Mll5, Mnat1, Mxd1, Ndufa13, Ntrk3, Pcbd2, Phtf1, Polr1a, Polr1b, Polr1c, Polr1d, Polr1e, Setd2, Sfxn4, Sin3a, Taf1, Tbp, Zfp467,	42
**GO.0006378**	mRNA polyadenylation	0.000197	Cpsf1, Cpsf2, Fip1l1, Papola, Wdr33	5
**GO.0051171**	regulation of nitrogen compound metabolic process	0.000198	Aipl1, Bloc1s2b, Brwd3, Cdk5, Chd2, Ctnnb1, Cux1, Dmrt2, Duoxa1, Dynll1, Ercc2, Ercc3, Gfi1b, Gtf2a1, Gtf2a2, Gtf2b, Gtf2e1, Gtf2e2, Gtf2h3, Gtf2h4, Gtf2h5, Hdac1, Klf1, Mll2, Mll5, Mnat1, Mxd1, Ndufa13, Ntrk3, Papola, Pcbd2, Phtf1, Polr1a, Polr1b, Polr1c, Polr1d, Polr1e, Ppp4c, Rbm20, Setd2, Sfxn4, Sin3a, Taf1, Tbp, Zfp467, Znrd1, Zxdc	47
**GO.0050794**	regulation of cellular process	0.000201	Aak1, Aipl1, Arl1, Bbs12, Bloc1s2b, Brwd3, Cacna2d1, Cacnb1, Cd1d2, Cdh2, Cdk18, Cdk5, Cdk5r1, Cetn2, Chd2, Crcp, Crhbp, Ctnnb1, Cux1, Dmrt2, Duoxa1, Dynll1, Epha8, Ercc2, Ercc3, Gab1, Gfi1b, Golga4, Gpr149, Grb2, Gtf2a1, Gtf2a2, Gtf2b, Gtf2e1, Gtf2e2, Gtf2h3, Gtf2h4, Gtf2h5, Hdac1, Ip6k2, Itih2, Kcnj8, Klf1, Ltbp3, Mll2, Mll5, Mrgprb1, Mxd1, Myo1d, Ndfip2, Ndufa13, Papola, Pcbd2, Phtf1, Polr1a, Polr1b, Polr1c, Polr1d, Polr1e, Ppih, Ppp4c, Ptpn11, Ptprr, Rbm20, Sbds, Setd2, Sfxn4, Sin3a, Snap25, Snx17, Soga3, Stfa2l1, Stx1a, Syt1, Taf1, Tbp, Txlna, Vrk1, Xpc, Zfand5, Zfp467	83
**GO.0010468**	regulation of gene expression	0.000407	Actg2, Bloc1s2b, Brwd3, Cdk5, Chd2, Ctnnb1, Cux1, Dmrt2, Dynll1, Ercc2, Ercc3, Gfi1b, Gfpt1, Gtf2a1, Gtf2a2, Gtf2b, Gtf2e1, Gtf2e2, Gtf2h3, Gtf2h4, Gtf2h5, Hdac1, Klf1, Mll2, Mll5, Mnat1, Mxd1, Ndfip2, Ndufa13, Papola, Pcbd2, Phtf1, Polr1a, Polr1b, Polr1c, Polr1d, Polr1e, Rbm20, Setd2, Sfxn4, Sin3a, Taf1, Tbp, Zfp467, Znrd1, Zxdc	46
**GO.0031124**	mRNA 3 -end processing	0.000488	Cpsf1, Cpsf3, Fip1l1, Papola, Wdr33	5
**GO.0006367**	transcription initiation from RNA polymerase II prom	0.000677	Gtf2a1, Gtf2a2, Gtf2e1, Gtf2e2, Taf7	5

GO ID	Pathway	FDR	Genes	Count
**GO.0010556**	regulation of macromolecule biosynthetic process	0.00111	Bloc1s2b, Brwd3, Cd1d2, Cdk5, Chd2, Ctnnb1, Cux1, Dmrt2, Dynll1, Ercc2, Ercc3, Gfi1b, Gtf2a1, Gtf2a2, Gtf2b, Gtf2e1, Gtf2e2, Gtf2h3, Gtf2h4, Gtf2h5, Hdac1, Klf1, Mll2, Mll5, Mnat1, Mxd1, Ndufa13, Ntrk3, Pcbd2, Phtf1, Polr1a, Polr1b, Polr1c, Polr1d, Polr1e, Setd2, Sfxn4, Sin3a, Taf1, Tbp,	43
**GO.0008150**	biological_process	0.00232	Aak1, Actg2, Arhgef10l, Bbs12, Bloc1s2b, Brwd3, Cacna2d1, Cacnb1, Ccdc124, Ccdc66, Ccdc87, Cd1d2, Cdh2, Cdk18, Cdk5r1, Cetn2, Chd2, Cpsf1, Cpsf3, Cpsf4, Crcp, Crhbp, Cstf1, Cstf2, Ctnnb1, Cux1, Cyp21a1, Dmrt2, Duoxa1, Dusp13, Epha8, Fam109a, Fbxl3, Fip1l1, Gab1, Gfi1b, Gpr149, Gtf2a2, Gtf2b, Gtf2e1, Gtf2e2, Gtf2h5, Hdac1, Iqsec1, Itih2, Klf1, Lamb2, Ltbp3, Mettl21d, Mettl6, Mis18bp1, Mll2, Mll5, Mrgprb1, Mrpl14, Mrpl15, Mxd1, Nat6, Ndfip2, Ndufa13, Ndufab1, Ndufb2, Ndufb5, Ndufb6, Ndufv2, Neu3, Papola, Pcbd2, Phtf1, Polr1a, Polr1b, Polr1c, Polr1d, Polr1e, Polr2e, Polr2f, Polr2h, Polr3b, Polr3c, Polr3e, Polr3f, Polr3h, Polr3k, Ppih, Prpf4, Ptpn11, Rbm20, Sbds, Scrn3, Setd2, Sin3a, Slc25a43, Snap25, Soga3, [illegible]	108
**GO.0070897**	DNA-templated transcriptional preinitiation complex	0.00285	Gtf2a2, Polr1e, Taf7	3
**GO.0002252**	immune effector process	0.00287	Crcp, Kcnj8, Mll5, Polr3b, Polr3c, Polr3d, Polr3e, Polr3f, Polr3h, Polr3k, [illegible]	11
**GO.0031326**	regulation of cellular biosynthetic process	0.00367	Bloc1s2b, Brwd3, Cd1d2, Cdk5, Chd2, Ctnnb1, Cux1, Dmrt2, Dynll1, Ercc2, Ercc3, Gfi1b, Gtf2a1, Gtf2a2, Gtf2b, Gtf2e1, Gtf2e2, Gtf2h3, Gtf2h4, Gtf2h5, Hdac1, Klf1, Mll2, Mll5, Mnat1, Mxd1, Ndufa13, Ntrk3, Pcbd2, Phtf1, Polr1a, Polr1b, Polr1c, Polr1d, Polr1e, Setd2, Sfxn4, Sin3a, Taf1, Tbp,	43
**GO.0001701**	in utero embryonic development	0.00651	Ctnnb1, Ercc2, Gab1, Grb2, Klf1, Mll2, Ptprr, Sbds, Setd2, Sin3a, Zfand5, mt-[illegible]	12
**GO.0006397**	mRNA processing	0.00708	Cpsf1, Cpsf3, Cpsf4, Cstf1, Cstf2, Fip1l1, Papola, Ppih, Prpf4, Rbm20, [illegible]	11
**GO.0032728**	positive regulation of interferon-beta production	0.00721	Polr3b, Polr3c, Polr3d, Polr3f	4
**GO.0048731**	system development	0.0142	Actg2, B2m, Baat, Bloc1s2b, Cad, Casq1, Ccdc66, Cdh2, Cdk5, Cdk5r1, Chd2, Cps1, Ctnnb1, Cux1, Dmrt2, Duoxa1, Dynll1, Epha8, Ercc2, Ercc3, Gab1, Gfi1b, Golga4, Gpr149, Grb2, Hdac1, Klf1, Lamb2, Ltbp3, Mll5, Mnat1, Ndufv2, Plce1, Ptpn11, Ptprr, Rbm20, Sbds, Setd2, Sin3a, Snap25,	43
**GO.0006379**	mRNA cleavage	0.0151	Cpsf1, Cpsf2, Wdr33	3
**GO.0002376**	immune system process	0.0195	B2m, Cd1d2, Chd2, Crcp, Ctnnb1, Ercc2, Gfi1b, Kcnj8, Klf1, Mll5, Polr3b, Polr3c, Polr3d, Polr3e, Polr3f, Polr3h, Polr3k, Ptpn11, Sbds, Sin3a, Sos1, [illegible]	23
**GO.0061198**	fungiform papilla formation	0.0285	Ctnnb1, Hdac1	2
**GO.0018107**	peptidyl-threonine phosphorylation	0.0397	Cad, Cdk5, Taf1, Vrk1	4
**GO.0006996**	organelle organization	0.0418	Arl1, Bbs12, Bloc1s2b, Brwd3, Casq1, Ccdc87, Cdk5, Cetn2, Chd2, Ctnnb1, Dynll1, Fam109a, Gtf2h4, Hdac1, Iqsec1, Klf1, Mis18bp1, Mll2, Mll5, Mrpl15, Polr1b, Sbds, Setd2, Sin3a, Snap25, Stx1a, Taf1, Tomm5, Vamp2, Vrk1, Xpc, Zmpste24	32
**GO.0017158**	regulation of calcium ion-dependent exocytosis	0.0466	Cdk5, Stx1a, Syt1, Vamp2	4

Supplementary Table 3. KEGG pathway analysis of 113 genes correlated with tumor volumes. *P*< 0.01.

Pathway ID	Pathway description	Count	FDR
3020	RNA polymerase	17	1.23E-27
240	Pyrimidine metabolism	19	1.29E-19
3022	Basal transcription factors	14	6.57E-18
5169	Epstein-Barr virus infection	19	3.17E-14
230	Purine metabolism	18	3.34E-14
4623	Cytosolic DNA-sensing pathway	12	9.62E-13
1100	Metabolic pathways	33	1.88E-09
3420	Nucleotide excision repair	8	2.57E-08
190	Oxidative phosphorylation	11	8.35E-08
3015	mRNA surveillance pathway	9	4.44E-07
5203	Viral carcinogenesis	11	4.78E-06
4721	Synaptic vesicle cycle	4	0.0186
5211	Renal cell carcinoma	4	0.0209
250	Alanine, aspartate and glutamate metabolis	3	0.0312

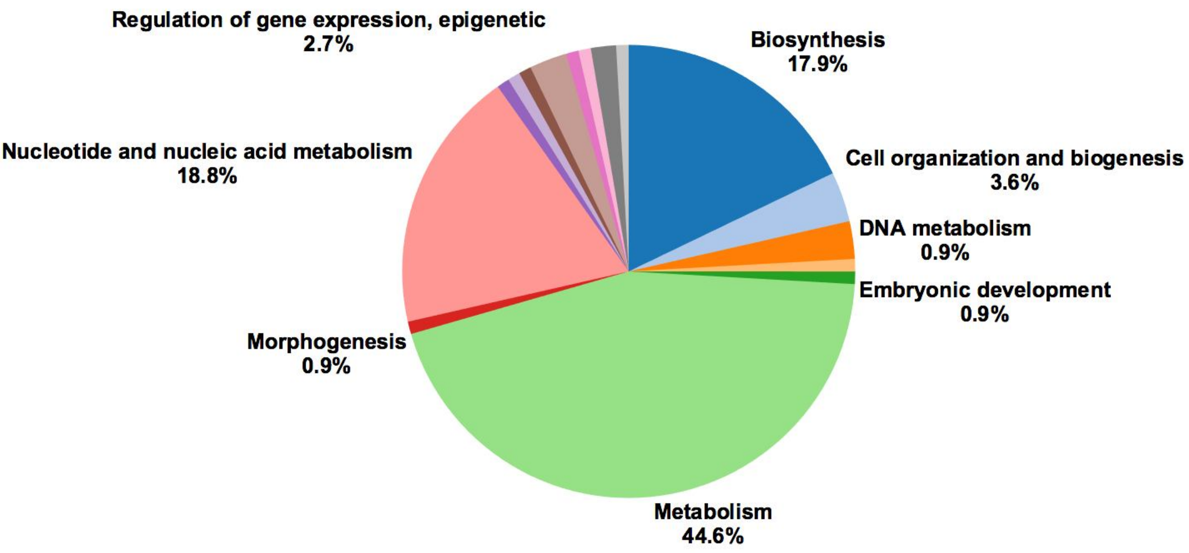

Supplementary Figure 1. Summary of gene ontology (GO) analysis of 113 genes correlated with tumor volumes. $P < 0.01$.
